# Immunomodulation with Recombinant Interferon-γ1b in Pulmonary Tuberculosis

**DOI:** 10.1371/journal.pone.0006984

**Published:** 2009-09-15

**Authors:** Rod Dawson, Rany Condos, Doris Tse, Maryann L. Huie, Stanley Ress, Chi-Hong Tseng, Clint Brauns, Michael Weiden, Yoshihiko Hoshino, Eric Bateman, William N. Rom

**Affiliations:** 1 Departments of Medicine and Environmental Medicine and Bellevue Chest Service, NYU School of Medicine, New York, New York, United States of America; 2 Division of Pulmonology, Department of Medicine, Health Sciences Faculty, University of Cape Town, Cape Town, South Africa; 3 Division of Immunology, Department of Medicine, Health Sciences Faculty, University of Cape Town, Cape Town, South Africa; Helmholtz Zentrum München/Ludwig-Maximilians-University Munich, Germany

## Abstract

**Background:**

Current treatment regimens for pulmonary tuberculosis require at least 6 months of therapy. Immune adjuvant therapy with recombinant interferon-γ1b (rIFN-γb) may reduce pulmonary inflammation and reduce the period of infectivity by promoting earlier sputum clearance.

**Methodology/Principal Findings:**

We performed a randomized, controlled clinical trial of directly observed therapy (DOTS) versus DOTS supplemented with nebulized or subcutaneously administered rIFN-γ1b over 4 months to 89 patients with cavitary pulmonary tuberculosis. Bronchoalveolar lavage (BAL) and blood were sampled at 0 and 4 months. There was a significant decline in levels of inflammatory cytokines IL-1β, IL-6, IL-8, and IL-10 in 24-hour BAL supernatants only in the nebulized rIFN-γ1b group from baseline to week 16. Both rIFN-γ1b groups showed significant 3-fold increases in CD4+ lymphocyte response to PPD at 4 weeks. There was a significant (p = 0.03) difference in the rate of clearance of *Mtb* from the sputum smear at 4 weeks for the nebulized rIFN-γ1b adjuvant group compared to DOTS or DOTS with subcutaneous rIFN-γ1b. In addition, there was significant reduction in the prevalence of fever, wheeze, and night sweats at 4 weeks among patients receiving rFN-γ1b versus DOTS alone.

**Conclusion:**

Recombinant interferon-γ1b adjuvant therapy plus DOTS in cavitary pulmonary tuberculosis can reduce inflammatory cytokines at the site of disease, improve clearance of *Mtb* from the sputum, and improve constitutional symptoms.

**Trial Registration:**

ClinicalTrials.gov NCT00201123

## Introduction

*Mycobacterium tuberculosis (Mtb)* infects one-third of the world's population, resulting in 9.2 million active cases per year [1]. Since the Directly Observed Therapy (Isoniazid, Rifampin, Pyrazinamide, Ethambutol 6 month Short Course, DOTS) approach is highly successful, the World Health Organization has set the goal of achieving 85% adherence, and the Global Plan to Stop TB has a vision of halving the prevalence and mortality of tuberculosis by 2015 [2]. Transgenic knockout models of mycobacteria-exposed mice demonstrate that interferon-γ and its signaling intermediates are critical to host defense [3], [4]. In humans, mutational defects in the interferon-γ receptor, the cytokine IL-12, or antibodies to interferon-γ result in disseminated mycobacterial infection [5], [6].

Sputum culture positive at two months, cavitation on chest radiography, being underweight, and bilateral pulmonary involvement increases the risk of treatment failure and/or relapse [7]. Responses of whole blood PBMC to PPD and other mycobacterial antigens are reduced in active tuberculosis probably due to suppressor T cells (Tregs ) or cytokines, e.g. IL-10 or TGF-β [8], [9]. Bronchoalveolar lavage (BAL) of patients with pulmonary tuberculosis has shown increases in inflammatory cytokines and percent CD4+ cells compared to uninfected controls, and in advanced, cavitary tuberculosis markers of effective immunity are reduced. Nitric oxide (NO) has been identified as a mechanism of mycobacterial killing, while mycobacteria can also be eliminated by autophagy, apoptosis, cytotoxic CD8+ cells, alpha defensins from neutrophils, and phagosomal-lysosomal rupture with subsequent cytosolic demise [10], [11], [12]. However, mycobacteria have evolved virulence factors to persist in macrophages, possibly disrupting the interferon-γ signaling pathways. We hypothesized that pharmacologic doses of rIFN-γ1b could augment the host immune response in TB, and to evaluate this we conducted a 4-month randomized, controlled clinical trial with rIFN-γ1b at 200 µg/day for three days/week with three arms: nebulized rIFN-γ1b or subcutaneous rIFN-γ1b with DOTS versus DOTS alone. End points were:: BAL cytokine release, treatment failure, sputum smear and culture conversion, and respiratory and systemic symptoms. We evaluated BAL cytokines,, mycobacterial and clinical response, immunological outcomes, and postulate mechanisms that rIFN-γ1b augmented to achieve an improved outcome.

## Methods

The protocol for this trial and supporting CONSORT checklist are available as supporting information; see Checklist S1 and Protocol S1.

### Study Subjects

Patients with pulmonary tuberculosis were recruited from April 2005 to December 2006 in the Division of Pulmonology at the University of Cape Town with the following inclusion criteria: sputum smear and culture positive for *Mycobacterium tuberculosis*, and bilateral cavitary tuberculosis. We randomized 96 patients who met the entrance criteria. Four of these individuals grew *Mtb* (BACTEC method) resistant to both isoniazid and rifampin and were thus ineligible due to MDR-TB, two patients had negative cultures for *Mtb*, and one patient had received a streptomycin-containing regimen. They had been randomized with 4 subjects in the DOTS plus subcutaneous IFN-γ, 2 in the DOTS alone group, and 1 in the DOTS plus nebulized IFN-γ group. All of the remaining 89 patients had *Mtb* cultured from their sputum and were drug-sensitive. Respiratory symptoms (cough, wheeze, dyspnea grade 2 or 3, sputum volume <10 ml) and night sweats (number/week) were reviewed at baseline and weekly during treatment by a blinded interviewer; oral temperature (fever defined as 38.5 °C) was recorded by the study nurse 3 times/week. IRB approval was obtained at NYU School of Medicine and the University of Cape Town; all study subjects signed informed consent in their native language. rIFN-γ1b (recombinant, InterMune Brisbane, CA) was nebulized with an AeroEclipse breath-activated nebulizer (which improves alveolar deposition), or was administered subcutaneously at 200 micrograms (excipient normal saline) three times per week over 4 months. All patients had weekly sputum smear and culture to 4 months and were followed up at 12 months with sputum smear and culture.

### Bronchoalveolar lavage

Bronchoalveolar lavage was performed at baseline and 4 months. Briefly, after local anesthesia with lidocaine, the bronchoscope was inserted via the nasal passage to the lower respiratory tract, wedged, and a 300-ml lavage was performed using 5, 20-ml aliquots of normal saline in each of three involved lung segments. The recovered fluid was pooled and filtered over sterile gauze, and a total cell count performed. A cytospin slide was stained with Diff-Quik and 500 cells counted to determine the cell differential. BAL supernatants were collected in RPMI over 24 hours at 10^6^ BAL cells/ml. Cytokines IL-1β, IL-6, IL-8, TNF-α, and IL-10 were measured by Luminex Beadlyte ELISA assay. Immunoblots for C/EBPβ were done as previously described [13], [14], [15].

### Systemic Response

Peripheral blood was obtained between 3–5 weeks for in vitro stimulation. Mononuclear cells (PBMC) isolated by Ficoll-Paque were cultured for 5 days in the absence or presence of staphylococcal enterotoxin B, Mumps antigen, or PPD and pulsed with BrDU during the last 24 hr. Cells were labeled with PerCP-Cy5.5-anti-CD4 and allophycocyanin-anti-CD25, fixed and permeabilized, then labeled with FITC-anti-BrDU in the presence of DNase I. Labeled cells were analyzed on a FACSCalibur, and lymphoblasts displaying PerCP-Cy5.5 were identified as CD4+. Proliferating cells were detected as CD25+BrDU+ lymphoblasts. The proliferation index is given by the %CD25+BrDU+lymphoblasts normalized by the %lymphoblast-sized cells in the CD4+ population in each culture.

### Radiographic Studies

Prior to entry into the study, tuberculosis patients had to have a posterior-anterior and lateral chest x-ray to evaluate for the presence of cavities. At randomization and 4 months, each participant had a chest CT-scan at the Groote Schuur Hospital using a Somatom Balance apparatus (Siemens Medical, Ehrlangen, Germany) and a single breathhold technique. The collimator was set at 3 mm standard cuts from the superior margin of the clavicles to the adrenal glands. The standard reconstruction algorithm was used for soft tissue and high frequency filter for the lung. The internal volume of each cavity was assessed using internal cross-sectional diameters.

### Statistical Analysis

The analysis, which included all 89 randomized and eligible subjects, was performed according to the intention-to-treat principle. Summary statistics (mean, median, standard deviation, and frequency distribution) were generated for baseline demographics and clinical presentations to characterize the study population. To compare the baseline characteristics between 3 treatment groups, one-way ANOVA or Kruskal-Wallis test was used for continuous variables, and Chi-square or Fisher's exact tests, for categorical variables (Table 1). The primary endpoint of the study was the time to smear conversion and culture conversion. Kaplan Meier curves were generated for the time to smear conversion by treatment groups. The log-rank test was used to compare the Kaplan Meier curves before 4 weeks. The analysis of culture conversion was done in the same way. For the change of symptom at 4 weeks, we averaged the results from the 3rd, 4th and 5th week data for each subject, and used one-way ANOVA and two sample t-test for 3 group and two group comparisons, respectively. For the analysis of cell differentials in BAL, functional assays, and cavity sizes, the paired t–test or Wilcoxon signed rank test was used to evaluate the change from baseline to 16 weeks within each treatment group (Table 2), and one-way ANOVA or Kruskal-Wallis test was used to compare the change from baseline to 16 weeks between treatment groups, as appropriate (Table 3). No adjustment for multiple comparisons was used and no missing data were imputed. All tests were two sided and p-values less than 5% were considered statistically significant. All analyses were conducted using SAS software.

**Table 1:** Baseline Table for Demographic Characteristics for the Three Arms.

	DOTS	NEBULIZED-rIFN-γ	SUBCUTANEOUS-rIFN-γ	p-value
**N**	30	32	27	
**Age years+/−SD**	32+/−11	34+/−10	35+/−13	0.55
**Gender**				
**Female**	37%	25%	19%	0.29
**Male**	63%	75%	81%	
**Race**				
**Black**	37%	41%	37%	0.73
**More than one**	63%	56%	63%	
**Asian/white**	0%	3%	0%	
**Cough**	96%	100%	100%	0.64
**Dyspnea = (2,3)**	93%	94%	92%	0.83
**Fever (oral T)**	46%	35%	42%	0.69
**Poor Appetite**	29%	45%	50%	0.24
**Wheeze**	68%	58%	54%	0.55
**Weakness**	50%	65%	62%	0.50
**Tiredness**	86%	77%	92%	0.29
**Night sweats (Nights/week)**	3.8+/−2.7	3.0+/−2.7	3.2+/−3.1	0.54
**Sputum Vol** <10 ml	29%	16%	12%	0.25
**WeightKg+/−SD**	54+/−9	56+/−10	55+/−7	0.71

**Table 2:** Bronchoalveolar Lavage Results.

	%			Cells/ml BAL Recovered (10e4)		
DOTS	Lymphocytes	Macrophages	Neutrophils	Lymphocytes	Macrophages	Neutrophils
Week 0	4 (2,8)	60 (17,84)	28 (10,82)	0.75 (0.26,1.50)	7.80 (2.58,12.75)	3.98 (0.96, 20.7)
Week 16	15 (6,25)	64 (44,72)	11 (3,31)	0.63 (0.40,1.20)	2.60 (1.98,4.68)	0.03 (0.12,2.00)
p-value	<0.01	0.13	0.01	0.90	0.09	<0.01
**NEBULIZED rIFN-γ**						
Week 0	5 (3,15)	60 (32,80)	24 (5,56)	0.84 (0.30,2.17)	7.43 (4.80,10.64)	2.10 (0.67,11.20)
Week 16	15 (8,34)	63 (40,84)	4 (2,16)	0.80 (0.52,1.60)	3.64 (1.98,7.20)	0.26 (0.14,0.82)
p-value	0.11	0.42	0.05	0.68	0.46	0.04
**SUBCUTANEOUS rIFN-γ**						
Week 0	6 (3,10)	62 (43,83)	30 (4,54)	0.56 (0.29,1.86)	7.26 (3.47,17.76)	3.59 (0.60,13.60)
Week 16	22 (13,33)	66 (54,77)	2 (2,9)	1.32 (0.58,3.22)	3.63 (1.90,6.50)	0.23 (0.08,0.52)
p-value	<0.01	0.94	0.02	0.09	<0.01	<0.01

* median (25–75 percentile).

**Table 3:** Proliferative Indices of CD4+ peripheral blood lymphocytes from cavitary TB patients after 3–5 weeks of treatment.

Treatment	N	SEB	MUMPS	PPD
DOTS alone	17	70.2±3.4	1.6±1.1	3.2±0.6
DOTS+ Nebulized IFN-γ	19	68.8±4.8	0.7±0.2	9.1±2.6
*p*, DOTS vs DOTS+ Nebulized IFN-γ		*0.825*	*0.475*	***0.041***
DOTS+ Subcutaneous IFN-γ	18	65.9±3.0	0.7±0.2	9.1±2.2
*p*, DOTS vs DOTS+ Subcutaneous IFN-γ		*0.359*	*0.444*	***0.017***

Proliferation indices (mean±SEM) were determined by flow cytometry (Fig. 3) following in vitro stimulation for 5 days with a T cell mitogen (SEB), Mumps antigen, or PPD at pretitered concentrations. Values given are those determined in the presence of antigen less that determined in the absence of antigen for the same specimen. The proliferation index in the absence of antigen was 0.2±0.4 for all specimens (N = 54). Significance *p* was determined using unpaired t tests at 95% confidence interval.

## Results

### Demographics

**Figure 1:**
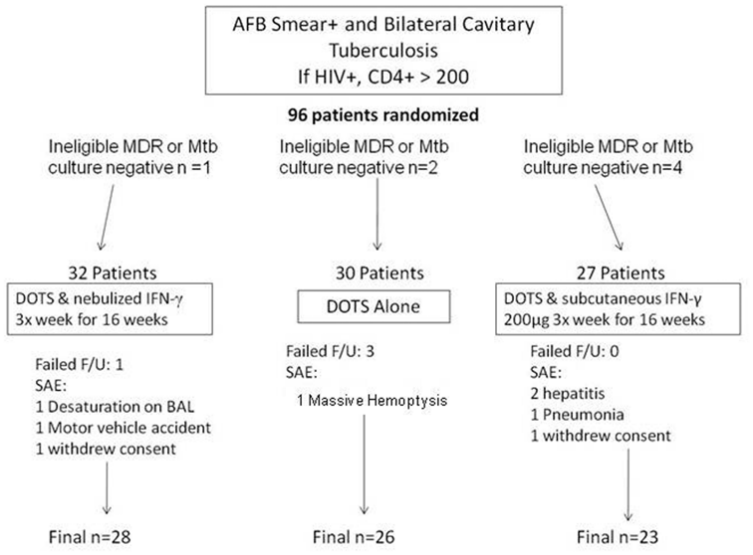
Flow Diagram showing number of study subjects screened, randomized to the three study groups n = 96, and who met inclusion criteria, n = 89).

There were 96 patients who were randomized and 89 who were eligible for the study. 10 patients did not complete the trial including 4 patients who were lost to follow-up, 2 who withdrew consent, and 4 who were withdrawn due to serious adverse events (SAE's): 2 hepatitis, 1 community acquired pneumonia, 1 hypoxia. Also 1 died from massive hemoptysis, and 1 died in a domestic accident (the SAE occurred after 16 weeks but before the end of their DOTS so were left in the analysis, Figure 1). The SAE's were not thought to be treatment-related based on Data Safety Management Board review although the hypoxia in the patient with emphysema occurred during the bronchoscopy and the procedure was terminated.

The mean age of the tuberculosis patients who were eligible for the study ranged from 32–35 years and did not differ among groups (Table 1). The 3 groups were closely matched for gender (63% to 81% male), and race (56–63% mixed) and symptoms. Eighty percent had a history of smoking. There were 6 patients who were co-infected with HIV-1 and their mean CD4+ was 253 cells/µL (all with CD4+>200 and randomized to DOTS 2, DOTS plus subcutaneous 1 and nebulized 3. At baseline, the three groups were comparable in respiratory and constitutional symptoms (Table 1).

### Bronchoalveolar lavage

There was a significant increase in median percent lymphocytes from baseline to 16 weeks in two of three groups in BAL (Table 2; DOTS 4% to 15%; rIFN-γ1b-SC 6% to 22%; rIFN-γ1b-NEB 5% to 15%, p<0.01 for DOTS and rIFN-γ1b-SC). There was no change in lymphocytes/ml BALF recovered. There was a significant decrease in median percent neutrophils from baseline to 16 weeks in all three groups in BAL (Table 2; DOTS 28% to 11%; rIFN-γ1b-SC 30% to 2%; rIFN-γ1b-NEB 24% to 4%), and a remarkable decline in neutrophils/ml BALF recovered in all 3 groups (DOTS 3.98 to 0.30×10^4^; rIFN-γ1b-SC 3.59 to 0.23×10^4^; rIFN-γ1b-NEB 2.10 to 0.26×10^4^). The percentage of macrophages increased slightly over the 16 weeks in all 3 groups. However, the macrophages/ml decreased 2-fold in all three groups (Table 2).

### Functional Assays

**Figure 2:**
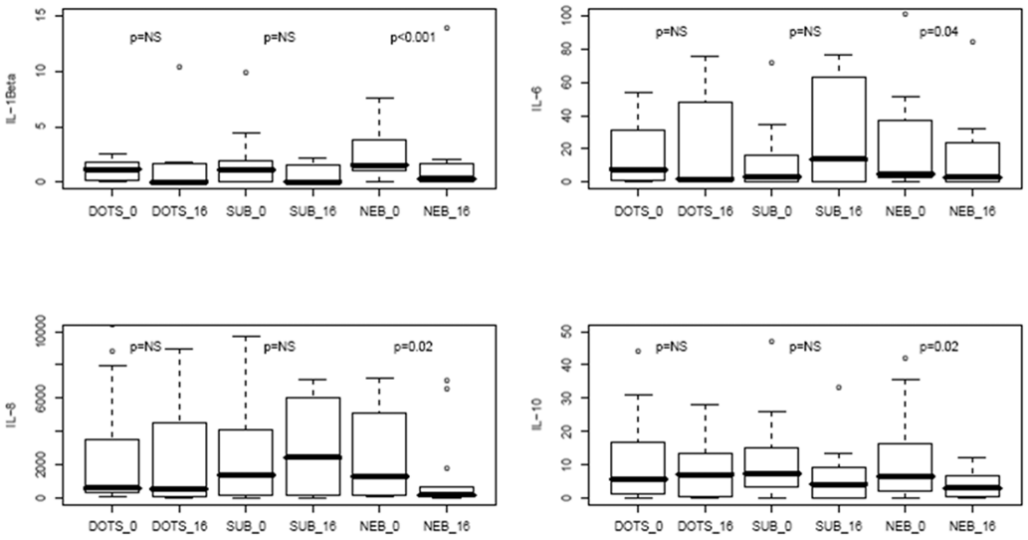
Reduction in inflammatory cytokines in 24 hour BAL supernatants by nebulized rIFN-γ1b comparing baseline to 16 weeks in ng/ml. A). IL-1β. DOTS or SC rIFN-γ plus DOTS, NS; NEB rIFN-γ1b plus DOTS, p<0.001. B). IL-6. DOTS or SC rIFN-γ plus DOTS, NS; NEB rIFN-γ1b plus DOTS, p<0.04. C). IL-8. DOTS or SC rIFN-γ plus DOTS, NS; NEB rIFN-γ1b plus DOTS, p<0.02. D). IL-10. DOTS or rIFN-γ plus DOTS, NS; NEB rIFN-γ1b plus DOTS, p<0.02.

There was a significant decline in inflammatory cytokines in 24 hour BAL supernatants in the nebulized rIFN-γ1b group only from baseline to week 16: IL-1β (p<0.001), IL-6 (p = 0.04), IL-8 (p = 0.02), and IL-10 (p = 0.02) (Figure 2).

**Figure 3:**
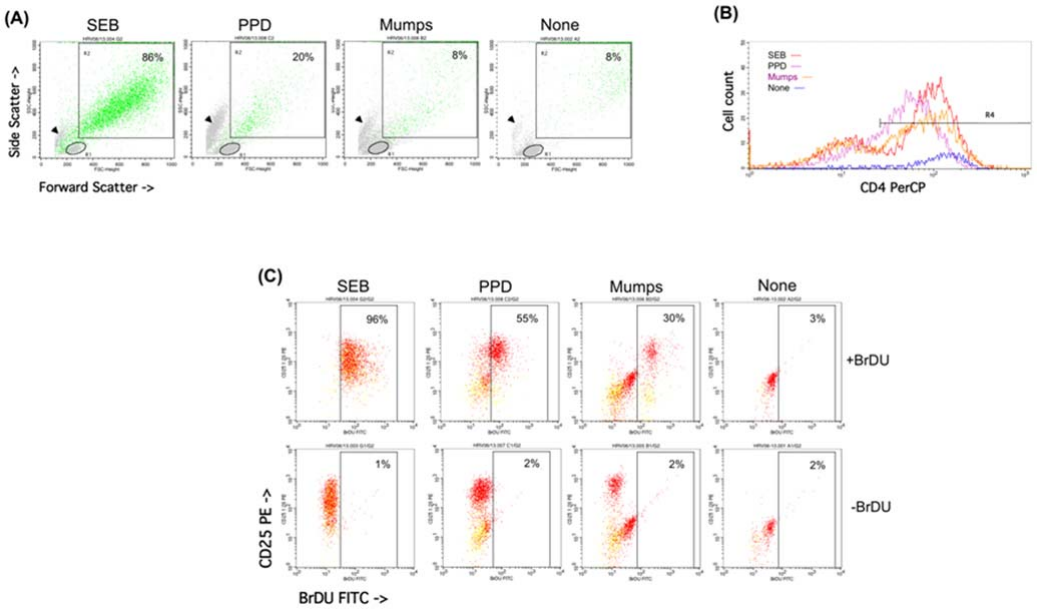
Detection of the CD4+ Proliferative Response from a Representative Patient. Peripheral blood mononuclear cells were cultured for 5 days, and pulsed with BrDU during the last 24 hr. Viable cells were labeled with PerCP-anti-CD4 and PE-anti-CD25, permeabilized and reacted with FITC-anti-BrDU in the presence of DNase, fixed and analyzed by FACS. (A) Lymphoblasts [R2] were discriminated from resting lymphocytes [R1] by forward and 90° angle (side) scattered laser light. Debris and necrotic cells are indicated by arrowheads. % lymphoblasts were given by the number of R2 cells divided by the number of R1+R2 cells. Approximately 10,000 events are shown in each dot blot. (B) CD4 intensity on R2 cells [R4] was used to select for CD4+ lymphoblasts. (C) Proliferating lymphoblasts which are displayed as green dots in (A). A total of 600

Peripheral blood lymphocytes showed a significant 2.8-fold increase in Th1 proliferation when stimulated with PPD but not SEB or MUMPS in vitro (Figure 3 and Table 3), demonstrating an enhanced antigen-specific systemic response after one month of both nebulized and subcutaneous rIFN-γ1b administration.

### Chest radiology

There was a striking improvement in cavity size as assessed by HRCT in all 3 groups between baseline and 16 weeks; however, the rIFN-γ1b groups did not differ from DOTS alone (Cavity in mm: DOTS 34+/−11 to 20+/−16; rIFN-γ1b-SC 39+/−24 to 29+/−24; rIFN-γ1b-NEB 34+/−13 to 18+/−17.

### Sputum Smear Conversion and Change in Symptoms

**Figure 4:**
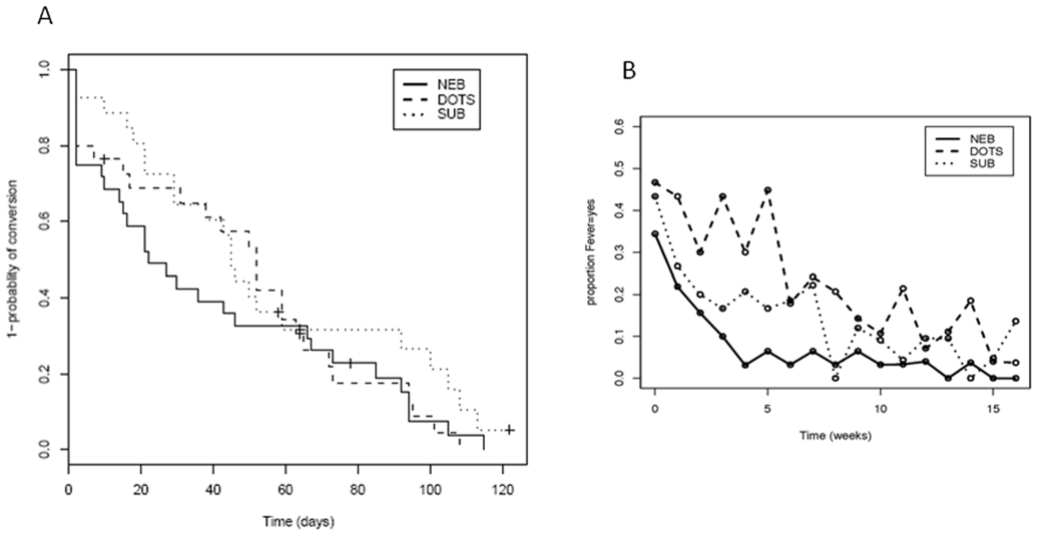
A. *M tuberculosis* sputum smear conversion. At 4 weeks, there was a higher *Mtb* smear conversion rate in the NEB rIFN-γ1b group compared to DOTS and subcutaneous IFN-γ1b plus DOTS (p = 0.03). At enrollment, all sputa were *Mtb* smear and culture positive. Y axis represents 1- probability of conversion in a Kaplan-Meier curve. B. Change in fever over 16 weeks comparing DOTS (dashed line) to DOTS plus nebulized rIFN-γ1b group (solid line). rIFN-γ1b significantly (p<0.05) reduced subjective fever at 4 weeks. Y axis represents proportion of patients having fever.

At 4 weeks, there was a significantly higher smear conversion rate in the nebulized rIFN-γ1b group compared to the DOTS control and subcutaneous rIFN-γ1b plus DOTS groups (60% versus 36% at 4 weeks, p = 0.03 for comparing Kaplan-Meier curves before 4 weeks, Figure 4A). A higher culture conversion rate at 4 weeks was also observed in the nebulized group compared to DOTS control and subcutaneous rIFN-γ1b plus DOTS (32% vs 18% at 4 weeks, p = 0.15). There was a significant reduction in fever in both rIFN-γ1b groups compared to control DOTS at 4 and 8 weeks (Figure 4B) and complaints of night sweats at 4 weeks in both rIFN-γ1b groups compared to DOTS control. Also, there was a significant reduction in the proportion with wheeze in both IFN-γ1b groups at 4, 8, and 12 weeks compared to DOTS control. In addition, at 12 weeks there was a significant reduction in cough in the groups receiving rIFN-γ1b compared to DOTS control. There was no statistically significant difference between treatment arms in the incidence of tiredness, poor appetite, sputum volume, or dyspnea. There was one treatment failure at 12 months in the DOTS alone group.

## Discussion

Immunoadjunctive therapy with nebulized rIFN-γ1b plus DOTS significantly reduced lung inflammatory cytokines in BAL supernatants at the 16 week time point. We treated five multiple-drug resistant tuberculosis patients with nebulized rIFN-γ1b while continuing their failing second-line regimens at a dose of 500 µg three times weekly for four weeks [13]. In all five patients, the sputum converted to negative, and symptoms improved. In order to investigate the mechanisms of these responses, we used nebulized rIFN-γ1b in eleven drug-sensitive tuberculosis patients, and found an increase in signaling molecules STAT-1, IRF-1 and IRF-9 [14]. In addition, nebulized rIFN-γ1b induced IP-10 downstream from these signaling molecules, but not inducible nitric oxide synthase (iNOS) [15]. There was a recruitment of lymphocytes and reduction in the neutrophil inflammation in the lung in all three groups, which manifested clinically as a dramatic resolution of the macrophage-neutrophilic alveolitis in the lower respiratory tract. Concomitantly with the increase in BAL lymphocytes, blood CD4+ cells doubled in their proliferative capacity to PPD or *Mtb* culture filtrate protein stimulation (data not shown) at the 4 week time point in both immunoadjunctive rIFN-γ1b groups of drug-sensitive pulmonary tuberculosis.

Immunoadjunctive therapy cleared *M tuberculosis* from the sputum and resolved the classic symptoms of fever, night sweats, wheeze, cough and malaise more rapidly than treatment with either DOTS or DOTS plus subcutaneous rIFN-γ1b. This finding suggests that nebulized rIFN-γ1b could reduce transmission due to more rapid clearance of *M tuberculosis* during the first 4 weeks. The subcutaneous rIFN-γ1b plus DOTS was not different from control since only nebulized rIFN-γ reaches the lower respiratory tract in pharmacological amounts to have a biological effect. All treatment groups experienced radiographic improvement with resolution of cavities by 16 weeks. Limitations of the study include the fact that this was an open-label, unblinded study without a placebo, and there was no group with rIFN-γ1b alone.

At 12 months there was only one study subject who had a treatment failure with culture positive sputum, and this occurred in the DOTS only control arm. TB Trials Consortium Study 22 comparing rifapentene and isoniazid once a week versus rifampicin and isoniazid twice a week reported bilateral pulmonary involvement and cavitation on chest radiograph were two risk factors (out of five) for treatment failure/relapse that occurred in 74/1004 (14.9%) of the TB patients [7]. Although our numbers were much smaller, we had no treatment failures/relapses among the adjunctive rIFN-γ1b groups.

This is the first major report on the efficacy of rIFN-γ1b in a randomized clinical trial. In the first study of nebulized versus subcutaneous rIFN-γ in humans, IP-10 mRNA was detected in BAL cells only in the nebulized patients and not subcutaneous rIFN-γ treated subjects [16]. This probably explains our finding that only nebulized rIFN-γ1b and not subcutaneous rIFN-γ1b was successful in clearing the sputum of *Mtb* and reducing the spontaneous release of inflammatory cytokines in 24 hour BAL supernatants. A pilot study of intramuscular rIFN-γ plus second-line anti-TB drugs over a 6-month period in 8 MDR-TB patients from Cuba showed sputum conversion over 1–3 months and marked improvement of radiographic abnormalities [17]. Giosue and colleagues nebulized IFN-α, a type I interferon, for 2 months as an adjunct in a randomized controlled clinical trial in 20 drug sensitive tuberculosis patients [18]. Improvements in *Mtb* number in the sputum at 7 days, reduced fever by 7 days, reduced number of nodules and area of consolidation on CT-scans at 2 months, and significant decreases in BAL fluid amounts at 2 months of IL-1β, IL-6, and TNF-α were noted in the IFN-α group [18]. In a clinical trial of interleukin-2 adjunctive therapy performed in Uganda, 110 TB patients were randomized to IL-2 subcutaneously twice/day for the first 30 days in addition to DOTS versus DOTS alone. Seventeen percent versus 30% sputum conversion, respectively, was observed at 4 weeks [19]. Tramontana and colleagues conducted a clinical trial of thalidomide in TB patients blocking TNF-α leading to enhanced weight gain and increased serum IFN-γ in vivo [20].

Microarray analyses of BAL cells from TB patients shows elevated cytokine IL-1β, adhesion molecule ICAM, TGF-β, apoptosis genes, and IFN-γ pathway genes [21]. Immunohistochemistry of *Mtb*-laden lungs show increased IFN-γ, IL-12, IP-10, and TGF-β in granulomas and areas with pneumonitis with reductions in IL-10 and IL-4 compared to controls [22]. BAL in Brazilian TB patients shows increased mRNA for TGF-βRI and II and IL-10 with increased protein for IFN-γ, IL-10, and bioactive TGF-β suggesting that TB has both stimulatory and counter-regulatory molecules that may ultimately down-regulate the Th1 response [23]. Th2 cytokines may be inhibited by antagonists such as IL-4δ2 that may be increased in TB [24]. PBMC responses from TB patients to *Mtb* in vitro are reduced, probably due to IL-10 which was increased in BAL cell supernatants. Gold and colleagues have reported low levels of Surfactant Protein A in BAL from involved segments of active TB along with increased IL-6, and reduced inhibitory C/EBPβ [25].

We noted that BAL lymphocytes increased in all three groups after 4 months of therapy. *Mtb* is suppressive of the T cell immune response, e.g. IFN-γ response to PPD-stimulated PBMC is depressed and TGF-β and IL-10 are increased at the beginning of TB treatment and return to normal levels only at the end of treatment 6–9 months later [26], [27]. In Gambian TB patients, T cell response to PPD from blood or pleural fluid was impaired pre-treatment, that changed to IFN-γ release after successful treatment or IL-4 after treatment failure; addition of IFN-α or IL-12 in vitro pre-treatment could alter the response to Th1 suggesting that immunotherapy could increase host defense against mycobacteria [28]. We showed significantly increased PBMC proliferative capacity to PPD in the nebulized and subcutaneous rIFN-γ1b groups within 3–5 weeks of immunoadjunctive therapy.

Inflammatory cytokines IL-1β, IL-6, IL-8, IFN-γ and TNF-α as well as anti-inflammatory cytokines IL-10 and TGF-β (and its TGF-βRI and RII) are elevated in BAL from active TB patients [29]. The inflammatory response in the lung in TB may include an increase in neutrophils, particularly in radiographically abnormal areas. Zhang and colleagues have previously reported that *Mtb* and its cell wall components stimulated IL-8 protein release and mRNA expression in vitro from alveolar macrophages [30]. In summary, the addition of nebulized recombinant interferon-γ1b to DOTS in a randomized, controlled clinical trial resulted in reduced BAL cytokines, more rapid clearance of *Mtb* from the sputum, improved symptoms, and reduced inflammatory macrophage-neutrophil alveolitis. These findings suggest that nebulized recombinant interferon-γ1b may have a role in adjunctive immune stimulation in patients with cavitary tuberculosis.

## Supporting Information

Checklist S1 CONSORT Checklist
(0.04 MB DOC)

Protocol S1 Trial Protocol
(0.36 MB DOC)
