# Supplementary material for: Immunomodulation with Recombinant Interferon-γ1b in Pulmonary Tuberculosis
Source: PLoS One. 2009 Sep 15;4(9):e6984. doi: 10.1371/journal.pone.0006984 (PMC2737621; doi:10.1371/journal.pone.0006984)
Supplement: Checklist S1 — CONSORT Checklist (0.04 MB DOC) [file pone.0006984.s001.doc]

CONSORT CHECKLIST (Ann Intern Med 2001; 134:663-694)

1. **TITLE/ABSTRACT** How participants were allocated to interventions

- “Randomized” Abstract line 4

1. **INTRO/BACKGROUND** Scientific background and explanation of rationale

- See Introduction that this is a “proof of principle” that the immune suppression identified in active pulmonary TB can be reversed by nebulized IFN-γ to the lung. BAL also can be used in South Africa to evaluate cytokines and inflammation.

1. **METHODS/**

**PARTICIPANTS** Eligibility criteria for participants and true setting and location where the data were collected.

- See Methods, lines 1-5

**4. INTERVENTIONS** Precise details of the interventions intended for each group and how and when they were actually administered

- See Methods, lines 12-14

**5. OBJECTIVES** Specific objectives and hypotheses

- See Introduction, last 5 lines

**6. OUTCOMES** Clearly defined primary and secondary outcome measures

- See Introduction, last 5 lines. The primary outcome measure was clearing AFB sputum smear and culture and treatment failure, and secondary outcomes were clinical signs, immunological response, and cytokines released by cells recovered by bronchoalveolar lavage.

**7. SAMPLE SIZE** How sample size was determined and any interim analyses or stopping rules

- The study was not powered for formal comparisons between treatment groups. Bilateral cavitary TB could be compared to a unilateral infiltrate where 32 subjects in DOTS vs DOTS plus nebulized interferon-γ treatment arms, a one-sided log rank test would achieve 80% power at a 0.05 significance level to detect a difference of 0.23 between 0.75 and 0.98, the expected proportions of patients who are relapse-free at 6 months in bilateral cavitary TB vs. unilateral infiltrate. These were the only conditions with data since no information was available at the outset on interferon-γ augmentation of the immune response.

**8. RANDOMIZATION/**

**SEQUENCE**

**GENERATION** Method used to generate random allocation sequence

- Patients with bilateral cavitary pulmonary TB were randomly assigned to DOTS plus subcutaneous interferon-γ vs. NIH grant and those in the NIH grant were randomly assigned to DOTS or DOTS plus nebulized IFN-γ with the intention of having 32 subjects in each group. Patients were originally to include Bellevue for one-fourth of the patients, but the study enrolled at only one site, Groote Schuur Hospital at the University of Cape Town. There were 6 individuals who were co-infected with HIV-1, and they were stratified to have 2 in each group. The NIH grant had only 2 groups, and the third group (DOTS plus subcutaneous IFN-γ) was added to obtain interferon-γ gratis. NIH required a separate protocol and randomization for this arm.

**9. RADOMIZATION/**

**ALLOCATION**

**CONCEALMENT** Method used to implement the random allocation sequence, clarifying whether the sequence was concealed until interventions were assigned

- Following the screening examination and determination of eligibility, the Biostatistician faxed the randomization to the study team. The PI was blinded throughout.

**10. RANDOMIZATION/**

**IMPLEMENTATION** Who generated the allocation sequence, who enrolled participants, and who assigned participants to their groups.

- The Biostatistician generated the allocation sequence, the first 2 authors (Drs. Dawson and Condos) enrolled participants, and the Biostatistician assigned participants to their groups.

**11. BLINDING** Whether or not participants, those administering the interventions, and those assessing the outcomes were blinded to group assignment

- The PI (William Rom, MD) was blinded to group assignment. The study nurse administered the nebulized or subcutaneous IFN-γ was not blinded. The study investigators completing questionnaires at clinic visits were blinded to group assignment.

**12. STATISTICAL**

**METHODS** Statistical methods used to compare groups for primary outcomes (AFB smears, culture of sputum) and secondary outcomes (clinical symptoms and signs; immunological parameters; BAL cell supernatant cytokines)

- See Methods, Biostatistics.

**13. RESULTS/PARTICIPANT FLOW**

- Figure 1 is a diagram describing flow of participants through each stage including number of participants randomly assigned, receiving nebulized or subcutaneous IFN-γ, and completing the study protocol. Those analyzed for primary outcome and listed and biostatistics discusses these in Results.

**14. RECRUITMENT**

- Dates defining recruitment were listed in Methods, lines 1-2, and follow-up was to 16 weeks for IFN-γ, 6 months for DOTS and 12 months for treatment failure.

**15. BASELINE DATA** Baseline demographic and clinical characteristics of each group

- See Table 1

**16. NUMBERS ANALIZED** Number of participants in each group included in each analysis and whether the analysis was “intention to treat”

- Figure 1 lists the number of participants in each group and the number randomized and meeting inclusion criteria (n=89) were analyzed as “intention to treat”.

**17. OUTCOMES AND**

**ESTIMATION** For each primary and secondary outcome, a summary of results for each group

- See results and Figures 2-4.

**18. ANCILLARY**

**ANALYSES** Address multiplicity by reporting any other analyses performed

- We address weight loss, tiredness, poor appetite, sputum volume, and dyspnea finding no significant differences (Results).

**19. ADVERSE EVENTS** All important adverse events or side effects in each intervention group

- See Figure 1 and Results, Demographics.

**20. DISCUSSION/**

**INTERPRETATION** Interpretation of the results, taking into account study hypotheses, sources of potential bias or imprecision, and the dangers associated, multiplicity of analyses and outcomes

- See Discussion.

**21. GENERALIZABILITY** Generalizability of the trial findings

- See Limitations in Discussion.

**22. OVERALL EVIDENCE** General interpretation of the results in the context of current evidence

- See Discussion
