# Supplementary material for: Immunomodulation with Recombinant Interferon-γ1b in Pulmonary Tuberculosis
Source: PLoS One. 2009 Sep 15;4(9):e6984. doi: 10.1371/journal.pone.0006984 (PMC2737621; doi:10.1371/journal.pone.0006984)
Supplement: Protocol S1 — Trial Protocol (0.36 MB DOC) [file pone.0006984.s002.doc]

Subcutaneous Interferon –γ for TB/HIV

PI William N. Rom MD, MPH

Co-PI Rany Condos MD

Protocol Version Date 03/18/05

**A. Specific Aims.**

The purpose of this study is to randomize TB patients to receive rIFN-γ 200 micrograms subcutaneously three times a week for 4 months on this protocol or to received treatment on one of the two randomized arms (aerosol IFN or placebo) of the concurrent funded study Host Response to TB and AIDS (NIH 059832). InterMune is providing the rIFN-γ free of charge for this study as well as for Host Response to TB and AIDS. Because the NIH funded protocol cannot be altered to add a third treatment arm, this protocol will provide the randomization of the additional patients to the subcutaneous arm to enable an unbiased comparison of the subcutaneous IFN to the aerosol and placebo arm. In particular, the hypothesis to be tested is whether type of administration (subcutaneous vs. aerosol) affects the end points of immune and fibrotic functions in pulmonary TB.

**The specific aims are:**

**Aim 1**. Randomize patients to receive subcutaneous IFN-gamma as described in this protocol vs. aerosol or placebo in NIH 059832.This study will be conducted in minimal to moderate pulmonary TB patients to evaluate the efficacy of 16 weeks of 200 mcg rIFN-γ 1b by subcutaneous injection three times a week plus Directly Observed Therapy Short Course (DOTS). We will allocate TB patients in a 2:1 randomization that allocates 2 patients to the NIH protocol for every 1 patient to this separate sub-study.

**Aim 2.** Evaluate BAL cells and PBMCs before and after rIFN-γ treatment to characterize the induction of a Th1 response.

**Aim 3**. Determine the *in vivo* molecular effects of rIFN-γ that relate to amelioration of fibrosis and tissue destruction in bilateral, cavitary TB by examining the TGF β system and pulmonary functional genomic studies.

**B. Background and Significance.**

Interferon-γ is a pleiotropic cytokine that has immune-modulating effects, e.g. activation of macrophages, enhanced release of oxygen radicals, microbial killing, enhanced expression of MHC Class II molecules, anti-viral effects, induction of the inducible nitric oxide synthase gene and release of NO, chemotactic factors to recruit and activate immune effector cells, downregulation of transferrin receptors limiting microbial access to iron necessary for survival of intracellular pathogens, etc. Genetically engineered mice that lack interferon-γ or its receptor are extremely susceptible to mycobacterial infection.

Recombinant IFN-γ was administered to normal volunteers and cancer patients in the 1980s through intramuscular and subcutaneous routes. There was evidence of monocyte activation, e.g. release of oxidants. In 1991, Jaffe and colleagues published a classic study of rIFNγ administration to 20 normal volunteers at The National Institutes of Health. First, they gave rIFN-γ 250 µg subcutaneously noting peak serum levels at 4 hour and a trough at 24 hour. Evaluation of respiratory epithelial lining fluid (ELF) from the BAL 1 hour, 4 hour, or 24 hour after subcutaneous rIFN-γ 250 µgm, could not detect any IFN-γ. IP-10 mRNA (a IFN-γ inductible chemokine) transcripts were present in blood monocytes recovered 1 hour after subcutaneous IFN-γ administration with faint transcripts in some individuals at 4 hour; in contrast, alveolar macrophages showed no IP-10 mRNA at any time points evaluated. Aerosolization of three different doses (250 µg, 500µg, or 1000µg) Monday, Wednesday, and Friday resulted in peak ELF levels at 1 hour after administration with the highest dose giving a three-fold higher peak *in vivo*. Levels of IFN-γ in the ELF were measured by a BIOELISA meaning that measured levels would be biologically active. No IFN-γ was detectable in the serum at any dose after aerosol delivery. The IP-10 mRNA transcripts were detected in alveolar macrophages 1 hour after the third aerosolization but not in blood monocytes. There were no local or systemic adverse effects including fever, nausea, etc nor decrements in pulmonary function following the aerosol delivery of IFN-γ. In contrast, participants receiving subcutaneous rIFN-γ experienced a spectrum of adverse events lasting from hours to days that included fever in all subjects, nausea, vomiting, headache, malaise, and local pain, induration, and erythema. Comparison of BAL total cells and cell differentials before and after 3 aerosol treatments found no significant differences between control and rIFN-γ in the aerosol. The levels of IFN-γ achieved by aerosolization were in excess of those needed to activate alveolar macrophages *in vitro* and the immune modulating effects were maximal at 1 hour. Martin and colleagues used 500 µg rIFN-γ as a dose for inhalation confirming up-regulation of IP-10 in alveolar macrophages and lack of adverse effects over 12 days in 5 subjects. They also noted a significant increase in BAL lymphocytes suggesting recruitment to the lung.

Holland and colleagues at the NIH added rIFN-γ subcutaneously (50 µg/m^2^) for 4-19 months to immunocompromised patients with refractory nontuberculous mycobacterial infection. They found a marked clinical improvement within 8 weeks of treatment when they added rIFN-γ to conventional anti-mycobacterial treatment. Clinical improvement included resolution of fever, bacteremia, adenopathy, organomegaly, increase in weight, albumin level, diarrhea, reduced skin lesions, clearing of sputum, marked reduction of ascites, and radiographic improvement.

We have completed several research studies in humans infected with *Mycobacterium tuberculosis*. We initiated BAL studies in patients with pulmonary TB finding increases in total cells and percent lymphocytes or neutrophils recovered. Supernatants and BAL fluid contained increased quantities of inflammatory cytokines (IL-1 β, IL-6, TNF-α, IL-8) but little or no IFN-γ. We then lavaged 30 patients and found a subgroup of 8 patients who had less clinical and radiographically advanced TB (smear-negative, noncavitary disease, culture-positive). They had an increase in BAL lymphocytes. Furthermore, BAL cells from these patients secreted IFN-γ but not IL-4, suggesting a Th-1 type lymphocytic response. Next we performed a pilot study adding aerosolized rIFN-γ to anti-mycobacterial therapy in patients with *M tb* resistant to isoniazid and rifampin (Multi-Drug Resistant TB). The 5 MDR-TB patients were on second line drugs for 5-24 months, were adherent to DOT, and had CT scans showing cavities with mild to moderate radiographic extent of disease. After 12 treatments (500 µg MWF X 4 weeks) there was clinical improvement (weight gain in three, stabilization in two), sputum smear conversion in all 5, and increase in time to detection of *M tb* by culture from 17 to 24 days (not statistically significant but an indication of decline in bacterial burden in the sample). CT scans showed significant improvement in one, stable or slightly improved in one, and no change (although there was reduction in cavitary lesions) in three. All five patients tolerated the therapy well. Adverse effects were minor and consisted of muscle aches (n=3) and cough (n=3). One patient had an episode of coughing associated with a 15% fall in peak expiratory flow rate, which responded well to nebulized albuterol. There were no recurrences of cough and none had wheezing or dyspnea. Third, we proposed and conducted a larger aerosol rIFN-γ clinical trial using each TB patient as their own control, assessing drug-sensitive TB, and evaluating signal transduction and gene expression in alveolar macrophages plus HIV-1 viral loads. In BAL cells from 10 TB patients, there was no STAT-1 or IRF-1 DNA-binding activity (both signaling molecules) 10 +/- 2 days post-antituberculosis treatment; however following 1 month of aerosol rIFN-γ in addition to the anti-tuberculosis drugs, 10/10 patients had increased STAT-1, IRF-1 and IRF-9 DNA binding activity in BAL cells from both radiographically involved and uninvolved lung segments. Symptoms and chest radiographs improved, and quantities of macrophage inflammatory cytokines and HIV-1 viral loads (in 5/5 co-infected patients) declined dramatically in the second BAL specimens. This study was not planned to have a control but Reviewers were anxious to see a randomized control study of rIFN-γ aerosol added to standard anti-mycobacterial versus anti-mycobacterial therapy alone.

InterMune sponsored several clinical trials to evaluate IFN-γ for infectious diseases. The MDR-TB clinical trial was entitled “A Phase II/III Study of the Safety and Efficacy of Inhaled Aerosolized Recombinant Interferon-γ 1b in Patients with Pulmonary Multiple Drug Resistant Tuberculosis (MDR-TB) Who have Failed an Appropriate Three Month Treatment.” This study enrolled 80 MDR-TB patients at several sites (Cape Town, Port Elizabeth, Durban, Mexico) and randomized them to receive aerosol rIFN-γ (500 µg MWF) or placebo for at least 6 months in addition to second line therapy. This clinical trial was stopped prematurely due to lack of efficacy on sputum smears, *M tb* culture, or chest radiograph changes. There were 15 deaths (10 rIFN-γ vs. 5 placebo, p=0.143). There were 5 in each group with hemoptysis as an adverse event. Review of all of the data provided by InterMune showed that the trial was biased toward the recruitment of patients with far advanced disease by chest radiograph. In the rIFN-γ group there were 2 minimal, 15 moderately advanced and 23 far advanced; the placebo group contained 1, 18, and 21 respectively. The far advanced patients were referred to as “salvage” patients. In our experience, these were the wrong types of patients to recruit because lung fibrosis, destruction and distortion make it difficult if not impossible for the aerosolized IFN-γ to reach areas of active infection, and second, with far advanced disease, there is little intact lung with macrophages to be modulated by an immune modulator. The other two trials on bronchiectasis (pulmonary MAC infection in non-HIV patients and cystic fibrosis) were also negative.

We have evaluated deposition studies in 7 TB patients receiving aerosol rIFN-γ. ^133^Xe equilibrium scanning, ^133^Xe washout, and ^99m^Tc-macro-aggregate albumin injection defined regional lung volume, ventilation and perfusion. We calculated that the lung dose averaged ~7% of the nebulizer charge (20-50 µg of rIFN-γ). Deposition was reduced only in those parts of the lung that appeared virtually destroyed on chest x-ray. We observed an overall increase in deposition in those areas with reduced ventilation including infiltrates. Aerosol mass median aerosol diameter (MMAD) was 3.2 microns as determined by cascade impaction. A significant proportion (44%) was particles greater than 5 µm. We used the Misty-Neb nebulizer for these studies; more recently, we have used the AeroEclipse and have documented a >2-fold decrease in oropharyngeal deposition (from 68% to 31%). In addition the MMAD was 2.2 microns-all consistent with a greater opportunity to achieve significantly more lung deposition and distribution.

Using the AeroEclipse nebulizer in the Host Response to TB and AIDS will allow us to reduce the dose to 200 mcg rather than 500 mcg and be the same as in this subcutaneous rIFN-γ trial. Respiratory tract deposition approximates 35 mcg using the AeroEclipse with a 250 mcg charge or 30 mcg using the former Misty-Neb nebulizer (original NIH Grant) with a 500 mcg charge.

A randomized clinical trial has been performed with aerosol IFN-gamma (Giosue et al 1998) on 20 patients wit drug sensitive TB for 2 months. The IFN-gamma group had more rapid reduction in sputum smear *Mtb* counts in the 1^st^ and 2^nd^ weeks, more rapid reduction of fever and increased HLA DR+ T Cells (p=0.009) in the IFN-gamma CT scans showed significant reduction for nodules (p=0.028) and consolidation (p=0.009) in the IFN-gamma group. The IFN-gamma group had more marked reduction in inflammatory cytokines. There were no adverse events, IFN-a/b and type I interferon’s have less of an immune modulating effect than IFN-gamma (type 2). The same group added 2 months of aerosol IFN-gamma to 7 patients with MDR-TB observing significance decreases in *M* *tb* in sputum smears and numbers of *M tb/*culture; HRCT scans were slightly improved.

Subcutaneous rIFN-gammaγ has been initiated as an adjunctive therapy in idiopathic pulmonary fibrosis (IPF) leading to a multi-center randomized clinical trial enrolling 330 patients. (Raghu 2004). The rationale is that rIFN-gamma reduces Transforming Growth Factor-beta and Connective Tissue Growth Factor. Ziesche et al reported a randomized, controlled study of 18 IPF patients treated with rIFN-gamma 200 mcg three times weekly for 12 months resulting in significant improvements in total lung capacity and PaO2. Using RT-PCR, there were also significant declines in TGF-beta and CTGF in transbronchial biopsies. These studies form the rationale of using TGF-beta and related molecules as endpoints in our clinical trial for the transbronchial biopsies to evaluate the healing process in our effort to prevent fibrosis from developing after pulmonary TB.

Raghu and colleagues reported a multi-center randomized controlled clinical trial of 330 IPF patients over 58 weeks using the same dose and found no effects on survival, lung function, or gas exchange. There was a 10% (rIFN-gamma) vs. 17% (placebo) survival advantage (p=0.08); among those with FVC > 62% predicted, 4% of rIFN-gamma treated patients died vs. 12% of placebo–treated patients (p=0.04). Assessment of biomarkers by Real-Time PCR showed increase for CXCL11(ITAC), an IFN-induced T cell chemoattractant and reductions in type I procollagen and PDGF-A(Strieter et al).

**Significance:** This clinical trial will compare data on subcutaneous IFN-gamma for TB to both aerosol and control subjects in Host Response to TB AIDS. Importantly rIFN-gamma 1b will be provided by InterMune free of charge for both clinical trials. We will compare results between delivery methods on alveolus and interstitium.

**C. Methods and Procedures**

**Specific Aim 1:** Randomize patients to receive subcutaneous IFN-gamma as described in this protocol vs. aerosol or placebo in NIH 059832.This study will be conducted in minimal to moderate pulmonary TB patients to evaluate the efficacy of 16 weeks of 200 mcg rIFN-γ 1b by subcutaneous injection three times a week plus Directly Observed Therapy Short Course (DOTS). We will allocate TB patients in a 2:1 randomization that allocates 2 patients to the NIH protocol for every 1 patient to this separate sub-study.

**Study objectives.** 1. Demonstrate that rIFN-γ therapy in patients with bilateral pulmonary TB with one or more cavities and positive AFB smear decreases the rate of failure/relapse from 25% (TBTC Clinical Trial) in the standard treatment group to 2% (Cohn et al, 1990) in the subcutaneous rIFN-γ treatment group. In addition, there will be an increased rate of closure of cavities, increased time to detection of *Mtb* in culture in the active treatment group, and more rapid conversion to culture negativity in the active treatment group.

2\ etc

**Study Endpoints.** 1. Compare sputum AFB smear, *Mtb* culture, and time to detection of Mtb in culture between the IFN-γ treatment and standard therapy groups. 2. Compare rate of closure of TB cavities by CT-scan at 1 month and 4 months between the IFN-γ treatment and standard therapy groups. 3. Compare BAL cell differentials and PBMC in flow cytometry to identify reduction in inflammation and more vigorous immune response at 1 month and 4 months between the IFN-γ treatment and standard therapy groups. 4. Compare expression of TGF-β and downstream molecules related to fibrosis and indices of healing in transbronchial biopsy specimens and BAL at 1 and 4 months between the IFN-γ treatment and standard therapy groups.

**Patient population.** We will screen 120 pulmonary TB patients. With the expectation that 96 patients will complete the clinical trial. The study population will consist of 96 evaluable male and female patients ages 18‑75 years old, who -? are smear- and sputum-culture-positive for *Mtb*. There will be 32 evaluable TB patients in this subcutaneous IFN-gamma arm. The patients must have pulmonary TB, with bilateral radiographic abnormalities and at least one cavity identified by chest radiography and/or CT scan. In patients with *Mtb*/HIV co-infection, we have traditionally treated the TB infection first, followed by initiating HAART immediately, following successful completion of the DOTS TB treatment. We expect to enroll three-fourths of the patients in Cape Town and one-fourth of the patients at NYU/Bellevue. TB patients should have ≤ 3 lobes involved (minimal to moderate disease).

### Inclusion criteria. Patients must fulfill all of the following criteria to be eligible for study admission:

1. Positive AFB smear on a sputum specimen obtained within 14 days prior to randomization.
2. To complete the study, the patient must have a positive sputum culture(Day 0, Day 8, and/or Day 14 to allow for sampling error) and not be MDR, i.e.; their *Mtb* organism must be sensitive to isoniazid and/or rifampin. Cultures are done with Lowenstein-Jensen medium, BACTC, or Becton-Dickinson MGIT bottles.
3. Age 18 through 75, inclusive.
4. HIV-1-positive patients must have CD4^+^ cells > 200/μL within 14 days prior to randomization. CD4+ cells 50-199/μL with CD4/CD8 ratio ≥ 0.3 allowed.
5. Women of childbearing potential may be entered if using effective contraception (barrier methods are acceptable) and having a negative serum beta-HCG within 48 hours prior to randomization.
6. The ability to understand and sign a written informed consent and comply with the requirements of the study.

## Exclusion criteria. Patients with any of the following will be excluded from the study:

1. Evidence of extrapulmonary tuberculosis on physical examination and/or radiologic studies.

1. HIV-1-positive patients, within the 30 days prior to randomization, who have had a serious opportunistic infection that would be expected to adversely affect their ability to participate in this clinical study. This includes but is not limited to: acute cryptococcal meningitis, encephalitis due to toxoplasmosis, acute *Pneumocystis carinii* pneumonia, disseminated herpes virus infections, and severe diarrheal illness requiring intravenous fluids.
2. HIV-1-positive patients who have ongoing serious opportunistic infections or HIV-related disease, including those listed above in exclusion criterion #4 as well as other chronic potentially life-threatening diseases associated with HIV infection, such as HIV-related malignancies, pulmonary or disseminated *M. avium* complex infection, or severe HIV-associated dementia.
3. HIV-1-positive patients on Highly Active Antiretroviral Therapy. (Due to toxic interactions with anti-TB drugs, especially rifampin or rifabutin).
4. Cancer, other than treated, localized basal cell carcinoma, cervical cancer, or localized Kaposi’s sarcoma.
5. History of life-threatening asthma or bronchospasm.

8. Pregnant or lactating women.

9. Pulmonary surgery within 90 days prior to randomization.

10. Initiation of new anti-retroviral therapy within 30 days prior to randomization.

11. HIV-1-infected patients should have completed therapy for any opportunistic infection within 30 days prior to randomization.

12. Chronic systemic corticosteroid therapy within 30 days prior to randomization or the requirement for corticosteroids during the trial.

13. Investigational therapy for *Mtb* within 90 days prior to randomization.

14. Treatment with rIFN-γ 1b within 90 days prior to randomization.

15. Cytotoxic drugs or immunosuppressive or immunomodulatory therapy within 90 days prior to randomization.

16. Investigational therapy for all other indications within 30 days prior to randomization.

17. Life-expectancy of ≤ 6 months.

18. Patients who, in the opinion of the Investigator, are not suitable candidates for enrollment or would not comply with the requirements for the trial.

1. History of symptomatic coronary artery disease, congestive heart failure, or valvular heart disease.
2. Evidence of chronic hepatic disease (bilirubin > 3mg/dl, and/or ALT, SGOT>3 times the normal hospital value, and/or alkaline phophatase >3times the normal hospital value) or chronic renal disease (creatinine > 3 mg/dl).
3. History of seizure disorder.

22. History of bleeding disorders or known clotting disorders.

**Study design.** This is a multicenter, randomized, study of a 16-week treatment regimen of rIFN-γ 1b delivered by subcutaneous injection in patients with pulmonary TB treated concomitantly with a stable Directly Observed Therapy Short Course (DOTS) regimen of antituberculous chemotherapy.

The active treatment group (n = 32 evaluable) will be randomly assigned to receive 200 μg of rIFN-γ 1b, three times per week in addition to IRPE (isoniazid, rifampin, pyrazinamide, ethambutol – Directly Observed Therapy Short Course [DOTS])

#### **Dosing regimen.** rIFN-γ will be administered via subcutaneous injection three times per week (e.g. Monday, Wednesday, and Friday) for a course of 16 weeks.

Dose: 0.5 mL from each of 2 vials will be mixed for a total volume of 1.0 mL as follows:

Active (200 μg dose level) -- 2 vials of rIFN-γ

**Drug administration.**

**For the subcutaneous injection**: For the first week (three doses), 0.5 ml from a single vial of active study drug will be drawn up into a 3 cc syringe and injected subcutaneously. Starting in the second week, 0.5 ml from each of two vials will be mixed to a total volume of 1.0ml and the full dose of 200 mcg of active drug will be injected subcutaneously.

**Screening evaluations.** Screening evaluations will be used to determine the eligibility of each candidate for study inclusion before any study medication is administered. All screening laboratory evaluations and the chest x-ray should be performed within the 14 days (unless otherwise specified) prior to study treatment.

1. Obtain written Informed Consent prior to performing any screening procedures

2. Complete medical history/review of systems including concomitant medications review

3. Complete physical examination including height, weight, oral temperature, and vital signs

4. Collection of sputum specimen for AFB smear and culture for *M. tuberculosis*

5. Routine clinical laboratory evaluations:

• Hematology (CBC with differential)

• Serum Chemistry Profile (electrolytes, BUN, creatinine, glucose, LDH, SGOT,ALT, alkaline phophatase, bilirubin)

• Urinalysis

• HIV–1 antibody test (with Western Blot confirmation, if positive)

• Serum pregnancy test (for women of child-bearing potential) within 48 hours

• Laboratory Evaluations for known or suspected HIV-positive patients to include CD4^+^ lymphocyte counts and viral loads

6. Chest x-ray

7. All study subjects will be counseled about HIV-1 testing and partner notification, according to local laws. Study subjects will have a p24 ELISA and Western blot confirmation, according to local procedures. CD4^+^ count and viral loads for HIV-1-positive study participants will be obtained. HIV-1-positive patients co-infected with *Mtb* who are on antiretroviral therapy will be excluded. TB patients who are diagnosed with HIV-1 infection will remain off HAART (antiretroviral) therapy for the first four months of the clinical trial. (NYU virologists typically withhold HAART until after TB treatment has been completed in accordance with CDC guidelines.)

8. Any other procedures deemed necessary to rule out exclusionary condition.

Eligible patients will be randomly allocated to the treatment group in equal proportions. Randomization will be stratified within country, by HIV status.

**Baseline/Day 0.** All Baseline clinical and laboratory evaluations must be performed prior to administration of study medication.

1. Collection of sputum specimen for AFB smear and culture for *M. tuberculosis*
2. Weight and Oral Temperature, vital signs
3. History since last visit with directed questionnaire (including concomitant medication review)
4. Abbreviated physical exam
5. CT Scan as per protocol
6. Whole blood in EDTA for serum, plasma, and extraction of DNA and total RNA from the buffy coat for research studies.
7. PPD – 5 tuberculin units. Induration in two directions read at 48 hr.

8. **Bronchoalveolar lavage (BAL) and Transbronchial Biopsy (TBBX).** A BAL using six 50-ml aliquots of normal saline instilled in radiographically involved sites (total 300 ml) will be performed under local xylocaine anesthesia. The recovered fluid will be filtered over sterile gauze, the volume recovered measured and a total cell count performed on a hematocytometer. 50,000-100,000 cells will be used for a cytospin slide to be stained with Diff-Quik. 500 cells will be counted to determine the cell differential. BAL cells will be pelleted and BAL fluid stored at –70°C. Transbronchial biopsies (2-3 per patient) will be performed in a non-cavitary, involved area of lung parenchyma after the BAL. Specimens will be snap frozen at –70°C and processed as described in Aim 3. Sterile sodium citrate will be added to the recovered fluid to a final concentration of 12.5 mM. BAL cells will be processed as described in Aims 2 and 3.

Each bronchoscopy will be monitored by an Attending Pulmonologist. Codeine (30 mg) is given IM prior to the procedure to reduce cough; the MD will monitor for nausea and cough. Bronchoscopy requires the patient to be NPO after midnight and the Attending MD will strictly enforce this. Consumption of food in the morning requires cancellation and re-scheduling the procedure. Lidocaine (3 ml of a 2% solution) is used in an aerosol spray for the back of the throat; lidocaine (2 ml of a 1% solution) is injected through the bronchoscope onto the vocal cords, followed by 2 ml of a 1% solution in the trachea, and 1 ml of a 1% solution in each main bronchus. The MD monitors lidocaine administration carefully for irregular heartbeat, lethargy, carpopedal spasm and seizures. No one will receive more than 30 ml = 300 mg of 1% lidocaine which is the maximum recommended safe dose according to the package insert. Midazolam (Versed) is given intravenously in 1 mg boluses to provide conscious sedation prior to the procedure; excessive midazolam can cause respiratory depression. Flumazenil (Romazicon), the benzodiazepine antagonist is kept at the bedside. The intravenous line is kept open and nasal O_2_ is administered continuously. The patient’s heart rhythm and oxygen saturation are monitored continuously before, during, and after the procedure. The BAL procedure uses 20 ml aliquots of normal saline are instilled followed by suction after each aliquot with a total of 160 ml in the radiographically involved site and eight 20 ml aliquots suctioned sequentially from the uninvolved site.

Transbronchial biopsies (2-3 per patient) will be obtained in a non-cavitary, involved area of lung parenchyma after the BAL. A potential complication of bronchoscopic biopsy is bleeding. This occurs in < 5% of procedures on the Bellevue Chest Service. If bleeding occurs, the bronchoscope is maintained in the bleeding site with suction, and epinephrine (3 cc aliquots of 1:20,000 dilution) may be injected through the bronchoscope. The pneumothorax risk for bronchoscopies involving transbronchial biopsies in our training program during the past 10 years is under 2%. Post-procedure fluoroscopy is done to rule out pneumothorax. If a pneumothorax is discovered, it will be managed according to its size and patient symptoms, if any. Small pneumothoraces which do not cause dyspnea or hypoxemia may be managed conservatively with observational and supplemental oxygen. Larger or symptomatic pneumothoraces will be managed by chest tube insertion with constant suction until the lung fully re-expands with no air leaks, usually requiring 1-3 days. The patient is monitored for vital signs and O_2_ saturation for 15 minutes over the next 2 hours following the procedure and is kept NPO for 3 hours for the gag reflex to recover. A chest exam for pneumothorax or wheeze, etc is performed post-procedure. Any wheeze will prompt treatment with aerosolized bronchodilator. The patient is called the next day for any post-procedure fever, which is treated with aspirin. Any further fever will be evaluated by the MD to rule out post-procedure pneumonia.

Following the IFN-γ aerosol, the patient will be monitored for any untoward reaction, e.g. fever, headache, malaise, nausea, vomiting. Any allergic reaction will result in prompt cessation of treatment.

Serious adverse events associated with medications and bronchoscopy requiring reporting include carpopedal spasm/seizure from lidocaine, respiratory depression from midazolam, bleeding in excess of 50 ml after transbronchial biopsy, post-procedure pneumonia requiring antibiotics, sustained arrhythmias, significant chest pain, and pneumothorax requiring a chest tube.

**CT technique for evaluation of bilateral parenchymal cavities.** All images will be acquired with either single- or multi-detector CT scanners: At Bellevue Hospital 1 Lightspeed QXI Multidetector CT and 1 CTI (sub-second) CT scanner (GE Medical Systems, Milwaukee, WI) will be used. At the Groote Schuur Hospital at the University of Cape Town 1 Somatom Balance (2001) (Siemens Medical, Ehrlangen, Germany) will be used. Studies will initially be performed without the use of intravenous contrast administration. A combination of thick and thin sections will be obtained in all cases in order to optimally evaluate pathologic changes. In each case, the external and internal diameters of all cavities will be assessed and the minimum and maximum wall thickness will be measured. The internal volume of each cavity will also be assessed using internal cross-sectional diameters. In addition, at Bellevue state-of-the-art workstations such as Advantage Windows (GE Medical Systems) or Vitrea (Vital Images) will be available for more sophisticated 3D renderings and volume determinations. Additional abnormalities to be assessed will include parenchymal nodules (in particular the presence of nodules with a tree‑in‑bud appearance indicative of active broncheolar inflammation), nodes (hilar and mediastinal), effusions (pleural and pericardial), bronchial abnormalities (bronchiectasis, bronchiolectasis, bronchial obstruction), and evidence of architectural distortion and/or volume loss in affected lobes.

**CT technique.** Patients will be placed in a supine position. Nonionic contrast (60%, 120 cc at 3 cc/sec) will be administered only as needed in select cases to clarify pathology. No oral contrast will be used. For patients with pleural effusion, one or two 5-mm images in the prone or ipsilateral lateral decubitus position will be obtained to evaluate for loculation.

|  | **CTI (single detector CT)** | **Siemens Somatom helical CT** | **Lightspeed (multidetector CT)** |
| --- | --- | --- | --- |
| **Collimator/Det** | 7.0 mm standard; additional select 3 mm sections through individual cavities | 8 mm standard: additional select 3 mm sections through individual cavities | 2.5 mm |
| **Pitch** | 1.6-2 | 1.6 - 2 | 6:1 (HS) |
| **Start point** | Superior margins of clavicles above lung apices | Superior margins of clavicles above lung apices | Superior margins of clavicles above lung apices |
| **End** | adrenal glands | Adrenal glands | adrenal glands |
| **Reconstruction thickness/interval** | 7.0 mm q 6 (entire chest); 3 mm q 2 through cavities) | 8.0 mm q 7 (entire chest); 3 mm q 2 through cavities | 5.0 mm q 4 (entire chest); 2.5 mm q 2 through cavities) |
| **Reconstruction algorithm** | Standard for soft tissue  High frequency E2 filter for lung | Standard for soft tissue  High frequency filter for lung | Standard for soft tissue  High frequency E2 filter for lung |
| Breath hold | single breath hold | Single breath hold | single breath hold |
| Window width | Standard mediastinal (450 HU) and lung windows (1500 HU) | Standard mediastinal (450 HU) and lung windows (1500 HU) | Standard mediastinal (450 HU) and lung windows (1500 HU) |
| PRINT | All images (12:1) | All images (12:1) | All images (12:1) |

**Study medication administration for 16-week treatment period.** Study medication will be administered three times per week (e.g. Monday, Wednesday, and Friday).

On the study treatment days, the following procedures will be performed:

- 1. Vital signs just prior to Study Medication Administration
  2. History since last visit with directed questionnaire (including concomitant medication review)
  3. Abbreviated physical exam
  4. Study Medication Administration (via nebulizer)
  5. Observe patient and take vital signs one-half hour post Study Medication Administration
  6. At the end of each week of treatment, a sputum specimen for AFB smear and culture for *M.*tuberculosis will be obtained
  7. At the end of each two-weeks of treatment, routine clinical laboratory evaluations will be performed.

At the end of one month of treatment:

- - 1. Bronchoalveolar lavage will be repeated.
    2. Whole blood in EDTA for serum, plasma, and extraction of DNA and total RNA from the buffy coat for research studies.
    3. HIV-1 viral load in HIV-1-positive patients.
    4. Antibody to IFN-γ to be done in collaboration with InterMune Inc. Repeat at 2 and 4 months. Antibody will be measured at time 0 or baseline since rare individuals may have antibody to gamma-interferon.

**Completion of study medication - week 16.** Administration of study medication will continue through week 16. At that time the following procedures will be performed:

1. Complete vital signs and laboratory work including hematology, serum chemistry, urinalysis, HIV antibody test (with Western blot confirmation if positive) and serum pregnancy test (for women of childbearing potential).

2. Safety measures at 20 weeks (hematology, serum chemistry (ALT and SGOT allowed to rise to 5 times baseline levels and alkaline phophatase to 3 times baseline level while on anti-tuberculous treatment), urinalysis).

3. Collection of sputum specimen for AFB smear and culture for *Mtb*.

4. Laboratory evaluations for HIV-1-positive patients: CD4^+^ count and HIV-1 viral load.

5. Chest x-ray and CT scan.

6. Weight, oral temperature and vital signs

7. Bronchoalveolar lavage and TBBX will be repeated.

8. Whole blood in EDTA for serum, plasma, and extraction of DNA and total RNA from the buffy coat for research studies.

9. PPD – 5 tuberculin units. Induration in two directions read at 48 hr.

**Study completion and follow-up to 2 years.** All study participants will be followed-up at 6, 12 and 24 months for health status, including:

1. Symptoms review and history since last visit with directed questionnaire

2. Physical examination

3. Obtain AFB smear and culture of sputum

4. Chest x-ray

A **failure** is AFB smear and/or culture positive for *Mtb* at 6 months. A **relapse** is AFB smear and/or culture for *Mtb* from 6 months to 2 years.

**Statistical considerations.**

Endpoints: Primary outcome measures:

1. The time from randomization to bacteriologic relapse, where bacteriologic relapse is defined as first positive culture following treatment.

2. The time from randomization to bacteriologic failure defined as persistent positive AFB smears at 5 months (ATS statement 1994) will be compared. These patients will be removed from the clinical trial for clinical evaluation.

3. The time from randomization to first negative AFB smear.

4. The time to detection of *Mtb* in culture (Epstein et al, 1998).

5. The time to culture negativity for *Mtb*.

Endpoints: Secondary outcome measures:

1. Proportion of patients with negative AFB cultures at 1, 2, and 4 months.

2. Change in maximal volume of cavities from chest radiographs and CT scans from baseline to 4 months.

3. Change in BAL cell differentials and mediators including the percent and absolute number of CD4^+^ and CD8^+^ lymphocytes from baseline to 4 months.

4. Change from baseline to 4 and 6 months in constitutional symptoms.

5. Change in baseline to 4 months in TGF-β and TGF-β-related molecules.

# *Sample size and power:* The total number of patients to be enrolled in the study is 96 with 32 patients randomized to treatment under this protocol with subcutaneous IFN-gamma and 64 rand to treatment with aerosol or placebo on the NIH protocol. It is anticipated that the study will accrue in 2 years. All subjects will be followed until the last subject enrolled has completed 2 years of follow-up. The primary analysis for the time to bacteriologic relapse will consist of estimation and confidence intervals for the hazard ratio for the the comparison of relapse free survival on subcutaneous IFN-gamma to the placebo group treated under the NIH protocol and for the comparison of the subcutaneous IFN group to the aerosol group under the NIH protocol.treatment groups. The study is not powered for formal comparisons between treatment groups; however, to define limits of the study, we present power calculations indicating the probability of obtaining statistically significant group differences in bacteriologic relapse rates. With a sample size of 64 subjects (32 in each treatment arm) a two-sided log rank test achieves 80% lower at a 0.10 significance level to detect a difference of 0.23 between 0.75 and 0.98, the expected proportions of patients who are relapse-free for 6 months in groups 1 (bilateral TB with cavity(s)) and 2 (unilateral infiltrate, no cavity), respectively. The results are based on the assumption of proportional hazards. The sample size calculation also allows for a 5% rate of subjects who are lost during follow-up. Subjects will be compared to controls (n=32) or aerosol rIFN-gamma (n=32) in Host Response to TB and AIDS. No adjustment will be made for the two comparisons of interest.

# *Analysis plan:* The study’s analysis will use an intent-to-treat approach. Subjects lost to follow-up before relapse and those not documented to have reached relapse at the end of the study will be considered censored. Analyses will be performed to describe baseline characteristics of randomized subjects and to compare rates of loss to follow-up between treatment arms.

The distribution of the time to bacteriologic relapse in each treatment arm will be estimated using the Kaplan-Meier method with comparisons based on the logrank statistic. The impact of baseline characteristics such as CT-scan findings, body weight, and race on the rate of relapse will be investigated using Cox proportional hazards models.

The time from randomization to first negative AFB smear, the time to detection of *Mtb* in culture, and the time to first negative culture for *Mtb* will be analyzed using methods for survival analysis as described for the time to bacteriologic relapse data using the logrank test to account for censoring. The effect of the stratification factor, HIV-1 co-infection, will also be examined by including it in the survival analysis model (Cox proportional hazards models) as a covariate.

Changes in the volume of chest cavities from baseline to 4 months will be compared between treatment groups using t test (or nonparametric test, whichever appropriate). The linear regression with adjustment for baseline characteristics and the randomization stratification factors will be used for the analysis. Subjects with both measurements will be included and the effects of missing values at the 4month observation will be examined. Changes from baseline in the percent lymphocytes and other BAL cells and cytokine detection will be assessed using the methods as described for the changes in volume of chest cavities. T test will be used to compare groups based on these endpoints after transformation to the log scale. Changes in TGF-β and related molecules will be analyzed similarly.

Changes in constitutional symptoms between baseline and 4 months will be compared between treatments using the Wilcoxon rank sum test.

The distribution of Tuberculin skin tests will be compared at baseline between cases and controls, and again after four months of treatment regimen using chi-square test (or Fisher Exact test). The PPD induration will also be evaluated, comparing baseline to four months in both groups.

*Randomization:* Subjects will be randomized using a 2:1 randomization that allocates 2 patients to the NIH protocol for every 1 patient to the separate subcutaneous rIFN-gamma in TB/HIV. The study patients will be stratified by HIV disease (HIV positive, HIV negative). The purpose of the stratification is to ensure balance between treatment arms with regard to HIV status at entry. There will be no limits on the number of subjects in each stratum. The analysis will also be stratified by NYU/Bellevue or University of Cape Town enrollment as characteristics of participants may differ at these sites.

**Assessment and Interpretation.** Will we recruit enough patients? To meet our sample-size needs, we must partner with TB research colleagues in order to enroll additional patients. We are proposing to join with Dr. Stanley Ress and colleagues at Groote Schuur Hospital at the University of Cape Town. We will process cells at Cape Town and ship them to NYC for flow cytometry and molecular studies. We are offering $200 per bronchoscopy ($600 per subject) for the inconvenience and time for the subject participation. This may also be an enticement in this patient population. Cape Town will also pay study subjects for their participation and bronchoscopies.

We chose 4 months of aerosol rIFN-γ treatment to extend the findings of our one-month trial and to have reasonable certainty of having a significant clinical effect. We extended to 4 months rather than 6 months in part due to the expense of rIFN-γ but, more importantly, this clinical trial, if efficacious, may provide an impetus to a larger clinical trial to shorten a 6-month DOTS regimen to 4 months with rIFN-γ. The TB Trials Consortium of 24 sites probably would be an appropriate forum for such a larger trial in drug-sensitive TB.

Are the endpoints failure/relapse, cavity size, and time-to-detection realistic? Failure has been defined as persistent smear/culture positive at six months and relapse is 12 or 24 months. High rates for failure/relapse have consistently been shown in minimal to moderate cavitary TB, with AFB positive sputum smears at two months. The choice of this difficult group of patients is significant, since this is the most important group of patients to demonstrate clinical efficacy of rIFN-γ. We are biasing the study against rIFN-γ with the expectation that it really does work. Other primary endpoints are key: time to *Mtb* culture negativity and time to detection, which increases prior to culture negativity.

**Specific Aim 2:** Evaluate bronchoalveolar lavage (BAL) cells and peripheral blood (PB) cells before and after rIFN-γ treatment to characterize the induction of a Th1 response. Cytokine profiles, phenotypic, activation and apoptotic markers on CD4^+^ and CD8^+^ T cells, proliferative response to tuberculosis proteins, inducible nitric oxide synthase (iNOS) expression and *Mtb* growth rate in alveolar macrophages will be determined. This specific aim will be performed by Investigator Doris Tse, PhD.

We will assess the immune status of study subjects before and after rIFN-γ treatment to characterize the induction of a Th1 response. We will analyze lymphocyte subset distribution as well as phenotypic, activation and apoptotic markers on CD4^+^ and CD8^+^ T cells in the BAL and PB. We will quantitate the levels of cytokines in BALF and plasma, as well as levels of cytokines secreted *in vitro* by BAL and PB cells, and we will evaluate the proliferative response of PB and BAL cells to TB antigens. We will determine the effect of rIFN-γ aerosol treatment on the expression of iNOS and correlate iNOS mRNA with apoptosis and *Mtb* growth rate in alveolar macrophages.

The PI and Dr. Rany Condos at NYU/Bellevue and Dr. Ress and colleagues at Groote Schuur Hospital at the University of Cape Town will perform the BAL/PB collections described in Aim 1. The handling and analyses of patient specimens for Aim 2 (Table 1) is modeled after the Adult AIDS Clinical Trials Group (ACTG) Immunology Study Guidelines. This strategy will ensure uniformity in processing, and limit the potential for disparity in the data to be obtained from specimens collected at different study sites. Assays with internal controls, e.g. standard curves, can be performed at either site using reagents/kits from the same supplier. Since it would be cost-prohibitive to institute a Quality Assurance Program between NYU and GSH for flow cytometric evaluation, cells will be labeled using fluorescent reagents from the same supplier at both sites, and analyzed by the CFAR Flow Cytometry core at NYUSOM. Likewise, assays that require special instrumentation (real-time PCR) or containment facilities (Mtb growth) will be conducted at the site of established expertise. Whenever possible, the same specimen will be stored in duplicate aliquots. Specimens will be shipped to laboratories in NYC or Cape Town by express freight (2-day international service). Lyophilized or fixed samples will be shipped at room temperature, plasma will be shipped in dry ice. Samples that are not fixed with formaldehyde will be shipped using biohazard containment procedures mandated by FAA/CDC guidelines.

### Endotoxin assay. BAL cells will be cultured at 37˚C in a 24-well plate in Dulbecco’s Modified Eagles’ medium (DME) supplemented with 2 mM glutamine and 10 μg/ml penicillin at a concentration of 10^6^ cells/ml. Supernatants will be collected over a 24-96 hr period in triplicate and lyophilized. All supernatants will be evaluated for the presence of gram-negative bacterial endotoxin with the E-toxate kit (Sigma). Uncontaminated samples should have <50 pg/ml endotoxin.

| **Table 1.** Handling and analyses of specimens collected at NYU/Bellevue Hospital and Groote Schuur Hospital | | | |
| --- | --- | --- | --- |
|  |  |  |  |
| **Specimen** | **On-site processing** | **Analyses** | **Laboratory** |
| Plasma | frozen at -70°C | BUN, cytokine assays | NYU/GSH |
| BAL fluid | concentrate & lyophilize | BUN, cytokine assays | NYU/GSH |
| BAL cell supernatant (spontaneous release) | lyophilize | endotoxin, cytokine assays | NYU/GSH |
| BAL & PB cells | label & fix | FACS | NYU |
| BAL & PB cells (antigenic response) | tissue culture | proliferation assay | NYU/GSH |
| BAL & PB cell supernatant (antigenic response) | lyophilize | cytokine assays | NYU/GSH |
| BAL cell RNA | lyophilize | real-time PCR | NYU |
| BAL cells | AM infection | *Mtb* growth rate | GSH |

**Lymphocyte subset, phenotypic, activation, and apoptotic markers.** Our goal is to assess alveolar and systemic lymphocytes (LC), in particular T cells, pre- and post-rIFN-γ aerosol treatment. The distribution of helper T cells (CD3^+^CD4^+^), cytotoxic/suppressor T cells (CD3^+^CD8^+^), gamma-delta T cells (CD3^+^γδTCR^+^), NK cells (CD56^+^), and B cells (CD19^+^) in the PB and BAL, as well as the coordinate expression of naïve/memory and early/late activation markers on CD4^+^ and CD8^+^ lymphocytes in PB and BAL will be determined. In addition, surface markers CXCR3 (receptor for IP10 and Mig) *vs* CCR3 (eotaxin receptor), and intracellular IFN-γ and IL‑4, characteristic of a Th1 *vs* Th2 host response will also be assessed (Sallusto 1998).

### The availability of high-speed 4-color flow cytometry has allowed us to phenotype T cells in BAL that are present at a considerably lower frequency compared to PB without the need to isolate alveolar lymphocytes (AL) by density gradient fractionation or adherence depletion (Raju 2001). Similar to industry standards for PB, we use cell size and granularity to discriminate BAL leukocytes during FACS analysis, and the purity of the AL population is evaluated by the presence of CD45^bright^CD14^-^ cells. Helper T cells can be identified as CD4^bright^ LC, and as many as three additional fluorescent labels can be used to study the coordinate expression of other surface molecules. Using this approach, we recently reassessed the surface phenotype of CD4^+^ lymphocytes in the lung to better define the role that different subsets may play in protecting the human host against *Mtb* (Raju et al, 2001). Most CD4^+^ LC from BAL, independent of whether it was collected from the involved or uninvolved lobe, co‑expressed CD29 and CD45RA. More remarkably, these CD45RA^+^ cells also expressed CD45RO.

For the proposed studies, PB and BAL specimens will be processed and labeled as previously described (Raju 2001), with minor modifications. BAL cells will be centrifuged in the cold and incubated in buffered NH_4_Cl for 7-10 min at 4°C to lyse erythrocytes. The remaining nucleated cells, mainly leukocytes, will be centrifuged and resuspended with 10 ml of prechilled cell dissociation buffer (Sigma) containing 5% fetal calf serum (CDB-FCS). The cell suspension is passed through a 70 μm cell strainer (Falcon) to remove aggregates, centrifuged and resuspended to 10^6^ lymphocytes per ml with autologous serum. Likewise, 1 ml of whole blood will be incubated with buffered NH_4_Cl for 7-10 min at 4°C to remove erythrocytes, washed with 10 ml of prechilled CDB-FCS, and resuspended in 1 ml autologous serum. Pre-mixed cocktails containing fluorescent monoclonal antibodies (mAbs) to multiple surface markers (Table 2) will be reacted with 100 μl of PB or BAL cell suspension on ice for 30 min. Irrelevant mAbs conjugated to the same fluorophores will be used to determine non-specific binding. Following surface labeling, cells will be washed with DPBS and fixed with 1% formaldehyde. We have established that removal of erythrocytes by NH_4_Cl, instead of FACSLyse (Raju et al, 2001), will allow labeled cells to be stored/shipped at room temperature for up to 72 hours without affecting the fluorophore intensity or % fluorescent cells detected by FACS. An aliquot of PB and BAL cell suspension will be incubated at 37°C for 4 h in the presence/absence of PPD and brefeldin-A (Sigma) to inhibit cytokine secretion. Following surface labeling with mAbs to CD4 and CD8, cells will be treated with FACS Permeabilizing Solution and reacted with mAbs to IFN-γ and IL-4 at room temperature for 30 min, washed with DPBS and fixed. All immunophenotyping reagents are available from Becton Dickinson Immunocytometry Systems (BDIS), Pharmingen, or R&D Systems.

Cells will be analyzed with a Becton-Dickinson FACS Calibur operated by the CFAR Flow Cytometry Core at NYUSOM. Dual Argon and HeNe ion lasers will be used for simultaneous 488 nm and 633 nm excitation. Emitted light will be detected by logarithmic amplification through barrier filters specific for the emission range of the different fluorophores used: 530/30 nm for fluorescein isothiocyanate (FITC), 585/42 nm for phycoerythrin (PE), 661/16 nm for peridinin chlorophyll protein (PerCP), and 675/20 for allophycocyanin (APC). Fluorescence spillover detected by inappropriate channels will be corrected by electronic compensation. Data acquisition and analyses will be performed using CellQuest software on a Macintosh G4 computer system. Forward scattered light (size) and 90° angle scattered light (granularity) at 488 nm will be used to exclude debris and select for lymphocytes or macrophages. When needed, emission intensities will be quantitated by normalizing against fluorescent bead standards (Molecular Probes).

| **Table 2.** FACS detection of phenotypic markers in BAL & PB lymphocytes | | | | |
| --- | --- | --- | --- | --- |
|  |  |  |  |  |
| **Tube** | **FITC** | **PE** | **PerCP** | **APC** |
| 1 | IgG | IgG | IgG | IgG |
| 2 | CD45 | CD56 | CD19 | CD14 |
| 3 | CD3 | TCRγδ | CD8 | CD4 |
| 4 | CD69 | CD25 | CD8 | CD4 |
| 5 | CD45RA | CD29 | CD8 | CD4 |
| 6 | CCR3 | CXCR3 | CD8 | CD4 |
| 7 | IFN-γ | IL-4 | CD8 | CD4 |
| 8 | dUTP | CD95 | CD8 | CD4 |

**Antigen-induced proliferation *in vitro*.** Mononuclear leukocytes will be isolated by Ficoll-Paque (Amersham Pharmacia) fractionation. Cells will be cultured in triplicate at 5x10^4^ cells/well for 5 days in RPMI supplemented with 10% autologous serum in the absence and presence of PPD (Accurate), short-term culture filtrate proteins from *Mtb*, and purified *Mtb* antigens, AraLam, Ag85, and ESAT6 (Colorado State University or Staten Serum Institute, Copenhagen, Denmark). We will also include TB-unrelated antigens such as streptokinase, tetanus toxoid, candida or Mumps antigen as indices of overall immune reactivity, and p24 as HIV-specific immune reactivity, pre- and post-rIFN-γ treatment. Cells cultured in the presence of phytohemagglutinin (PHA) or the superantigen streptococcal enterotoxin B (SEB) will serve as a positive control for TCR-mediated mitogenesis, while cells cultured in phorbol myristic acid and ionomycin will serve as a positive control for TCR-independent mitogenesis. Cells will be pulsed with BrDU during the last 24 h. Culture supernatants will be harvested and BrDU incorporation will be measured using a colorimetric immunoassay kit (Roche) according to supplier instructions. Likewise, the *in vitro* response of BAL mononuclear leukocytes to PPD, which is known to be upregulated in TB effusions compared to PB (Lukey 1996), will be assessed pre- and post-rIFN-γ treatment.

The *in vitro* reactivity of PBMCs from selected subjects will also be studied by flow cytometry, as described in Fig. 2, to determine if there are changes in the magnitude of the immune response to TB antigens mediated by CD4^+^ and CD8^+^ lymphocytes pre- and post-rIFN-γ treatment, and whether CD4^+^ and CD8^+^ T-cell proliferation is driven by endogenous IL-2 secretion.

### Cytokine assays. Serum samples will be frozen and stored at -70°C. BALF will be concentrated 20-fold on AMICON ultrafiltration membranes and lyophilized. Supernatants from 24-hr BAL cell cultures (spontaneous release) and supernatants from PB and BAL mononuclear cells cultured for 5 days in the presence of TB and non‑TB antigens will also be lyophilized.

### All samples will be assayed for Th1/Th2 and pro-inflammatory cytokines with a Cytometric Bead Array (CBA) kit from Pharmingen. Using this methodology, levels of 6 different cytokines, IFN-γ, IL-2, IL-4, IL-10, TNF-α, and IL-6, can be measured simultaneously on 50 μl of specimen. IL-8, IL-12, Mig, I-TAC, and IP10 will also be measured with enzyme-linked immunosorbent assay (ELISA) kits from Biosource International. These cytokines will allow us to gain additional insight into the mechanisms underlying rIFN-γ-mediated regulation/amplification of a TB-specific Th1 response. The sensitivity of the CBA assay is similar to that of the more conventional ELISA system. BUN values obtained on serum and BALF will be used to convert cytokine levels in BALF to pg/ml epithelial lining fluid using the urea dilution method.

### As needed, we will modify commercial ELISA kits with a highly sensitive fluorometric detection technique. β-galactosidase, which catalyzes the conversion of 4-methylumberlliferyl phosphate (MUG) from a non‑fluorescent substrate to a fluorescent product, will be used in place of horseradish peroxidase (HRP). A 70-fold increase in the sensitivity of detection was obtained for IFN-γ compared to conventional colorimetric methods. Prolonging the incubation time from 24 to 48 hours allowed increased production of fluorescent MUG product and, consequently, further increase in sensitivity. This modification will not only provide for lower limits of detection, an advantage for monitoring IFN-γ levels in plasma and BALF, but where only limited volumes of cell culture supernatants are available, it will allow us to determine the levels of all the cytokines that we propose.

### Inducible nitric oxide synthase expression. Molecular beacons are novel oligonucleotide probes that can be used to monitor the presence of specific nucleic acid sequences in solution (Tyagi & Kramer, 1996). Upon annealing to their targets, they undergo a conformational reorganization that restores the emission of an internally quenched fluorophore. Fluorescence increases as much as 900-fold when molecular beacons bind to their targets, obviating the stringent need to remove unhybridized probe inherent in conventional methods. Molecular beacons can discriminate targets differing by as little as a single nucleotide. The intensity of their fluorescence as a direct measurement of target concentration and their application in monitoring polymerase chain reactions (PCR) in real time has been established (Kostrikis et al, 1998).

### Total RNA will be isolated using an RNeasy Mini Kit (Qiagen) according to supplier instructions. A Superscript Preamplification System (Life Technologies) will be used to generate first strand cDNA from the total RNA with an oligo-dT primer. We have found that a combination of these procedures is optimal for isolating and quantitating mRNA from small numbers of cells. A suitable portion of this cDNA will be subjected to PCR amplification with forward and reverse primers for iNOS, GAPDH, and CD14, as shown in Table 3. Forty to sixty cycles of amplification (94˚C denaturation for 30 sec, 60˚C annealing for 60 sec, and 72˚C polymerization for 30 sec) will be performed in an ABI Prism 7700 spectrofluorimetric thermal cycler (Applied Biosystems), operated by the CFAR Virology Core at NYUSOM. The number of cycles that it takes for an exponential amplificaton reaction (measured as fluorescence intensity at the emission wavelengths, shown in Table 4) to enter the linear phase of synthesis is inversely proportional to the initial number of template molecules, and the concentration of the cDNA in the sample can be calculated using a standard (e.g., plasmid containing iNOS cDNA inserted into the pREP4 vector, genebank accession #L09210) amplified in a parallel reaction.

| **Table 3.** Sequences of primers used for RT-PCR | | |
| --- | --- | --- |
|  |  |  |
| **mRNA** | **Forward Primer** | **Reverse Primer** |
| iNOS | TGGCAGCATCAGAGGGGACC | GCAGGACCAGGGGGACCACTCGAA |
| GAPDH | GACCCTCACTGCTGGGGAGT | ACTGTGAGGAGGGGAGATTC |
| CD14 | GGTGCCGCTGTGTAGGAAAGA | GGTCCTCGAGCGTCAGTTCCT |

#### There is a distinct possibility that iNOS expression determined by real-time PCR can be skewed by heterogeneity in the leukocyte population in BAL cell samples collected pre- and post-rIFN-γ treatment. Even when iNOS mRNA is normalized against GAPDH mRNA, the expression of housekeeping genes (which is an effective control for RNA recovery and cDNA synthesis) is likely to vary amongst different cell types. Thus, we will quantitate CD14 mRNA as a means of monitoring AM-specific gene expression (Cario 2000). This will allow us to determine whether iNOS expression is upregulated in AMs pre- and post-rIFN-γ treatment, or whether overall iNOS activity in the infected lung segment is enhanced by an IFN-γ-mediated increase in the number of AMs.

| **Table 4.** Molecular beacons used for amplicon detection in real-time PCR | | |  | |  |
| --- | --- | --- | --- | --- | --- |
|  |  |  | |  | |
| **cDNA** | **Beacon sequence** | **Fluorophore** | | **Emission** | |
| iNOS | 5'-*CCGACG*GCTGGAAGCCCAAGTACG *CGTCGG*-3' | FAM | | 518 nm | |
| GAPDH | 5'-*GGACGC*GGTGGGGGACTGAGTGTG *GCGTCC*-3' | JOE | | 554 nm | |
| CD14 | 5'-*GGCAGG*TGTGAGCTGGACGATGAAGATTT*CCTGCC*-3' | TAMRA | | 583 nm | |
|  |  |  | |  | |
| Oligonucleotides are conjugated to the universal quencher DABCYL at the 5' end and the indicated fluorophores at the 3' end. When complimenary stem sequences (italics) in the hairpin structure dissociate upon annealing of the loop sequences to their targets, DABCYL is removed from the proximity of FAM, JOE, and TAMRA, thus allowing the fluorescence of these fluorophores to be detected at their respective emission wavelengths. | | | | | |

**Collaborative studies of *Mtb* growth in alveolar macrophages at the University of Cape Town.** Higher levels of apoptosis have been demonstrated in both CD4^+^ T cells and macrophages isolated from TB (Klingler et al, 1997; Hirsch et al, 2001). Excess pleural T-cell apoptosis was associated with high local levels of IFN-γ, suggesting that IFN-γ might enhance apoptosis at sites of active *Mtb* infection (Hirsch et al, 2001). We will investigate whether IFN-γ enhances or reduces apoptosis of macrophages and/or T-cell subsets and relate this to *Mtb* growth in macrophages. We will also determine if T-cell apoptosis is elicited via the Fas pathway. The current study would provide an opportunity to evaluate the effect of inhaled IFN-γ on BAL-derived macrophages and T cells. Firstly, the effect of IFN-γ-induced macrophage activation on mycobacterial growth rates will be evaluated utilising standard radiometric labelling assay as previously described (Passmore et al, 2001). [Adherent macrophages will be infected with *Mtb* H37Rv in the presence of 10% FCS and incubated at 37^0^C. On days 0 (90 minutes post-infection), 1, 2 and 4, the cells will be lysed using 0.25 % SDS. The cell lysate will be diluted 10-fold in 10% OADC Middlebrooks 7H9 broth (Difco), plated into a 96-well plate (100 μl.well^-1^; replicates of six per treatment), and pulsed with [^3^H]-Uridine (1 μCi.well^-1^; Amersham) for 10-12 days at 37^o^C. Bacterial cell-associated radioactivity will be measured using a liquid scintillation counter (Tricarb 4640, Packard)]. Secondly, the impact of IFN-γ on apoptosis of macrophages (CD3-CD14^+^) and T-cell subsets (CD3^+^CD4^+^ and CD3^+^8^+^) will be evaluated. An aliquot of PB and BAL cell suspension will be processed according to the ACTG protocol for TUNEL assay: cells will be reacted with a pre-mixed cocktail containing mAbs to CD4, CD8 and CD95 (Fas/APO-1), fixed and permeabilized, then incubated with terminal deoxynucleotidyl transferase and FITC-dUTP (Roche In Situ Cell Death Detection kit, Fluorescein). Results of both experimental approaches will be compared to those obtained with cells isolated from BAL from the control patient group not receiving inhaled IFN-γ therapy. These experiments should help resolve the important question of whether the beneficial effects of IFN-γ therapy on host protective immunity against mycobacterial infection may be related to apoptosis as a killing mechanism of *Mtb*.

**Assessment and critique.** Aim 2 allows us to obtain descriptive data on BAL/PBMC cells and subsets to relate to rIFN-γ. We can obtain functional data with cytokines, antigen response, and iNOS gene expression before and after rIFN-γ treatment. S. Ress will also have the important opportunity to investigate apoptosis in BAL macrophages and lymphocytes and relate this to *Mtb* growth. Sufficient BAL cells (25-30 x 10^6^) from involved segments of TB patients will be available to obtain these measurements. As a member of the ACTG Immunologists, Dr. Doris Tse meets and follows guidelines for cellular immunological studies and quality control. Dr. Tse collaborates with the NYU/Bellevue TB group and has a collaboration with Dr. Rom on evaluating the immune response to TB patients from a candidate TB vaccine.

We predict that a CD4 Th1 phenotype will develop and be maintained over the four-month course of rIFN‑γ aerosol treatment. Usually TB patients have BAL/PBMC that are hyporesponsive to *Mtb* antigens, and the prediction of rIFN-γ treatment is that this will resolve more rapidly in the treated group than those receiving DOTS standard treatment. Cytokine BAL levels will drop more rapidly in the rIFN-γ group. Lastly, iNOS will be higher in the rIFN-γ group and persist to the four-month data point. One critique is that tachyphylaxis will occur and no further cellular or molecular endpoint will persist; however, we predict that cellular as well as clinical endpoints will be more robust in the rIFN-γ treatment group.

# Specific Aim 3: Determine *in vivo* molecular effects of IFN-γ that relate to amelioration of fibrosis and tissue destruction and improved clinical outcome in bilateral, cavitary pulmonary tuberculosis by (a) examining the TGF-β system and (b) performing functional genomic studies.

**(a) Rationale for examining the TGF-β system.** As described in greater detail above, TGF-β is implicated in the immunopathology of TB and in the tissue damage of IPF. IFN-γ is critical for normal host defense against *Mtb*. IFN-γ therapy has been shown to have some effect against TB and IPF. The effect of IFN-γ against IPF correlates with antagonism of the TGF-β system, thus it is likely that the same will be true for at least some effects of IFN-γ therapy for TB. Importantly, molecular examples of IFN-γ antagonism of TGF‑β effects provide precedent for these studies.

Antagonism of TGF-β by IFN-γ includes suppression of genes induced by prior exposure to TGF-β and induction of genes suppressed by prior exposure to TGF-β. Many of the genes that can be suppressed by TGF-β and induced by IFN-γ, such as the chemokine MCP-1 (Kitamura, 1997), the adhesion molecule ICAM-1 (Xiao et al, 1996; Tomioka et al, 2000) and the MHC class II transactivator (Lee et al,1997; Nandan & Reiner, 1997; Piskurich et al, 1998), are up-regulated directly or indirectly by STAT-1, the primary mediator of transcriptional activation in response to IFN-γ (Darnell, 1997). Changes in expression of such genes will not distinguish between decreased effects of TGF-β and the response to IFN-γ *per se*; thus, we will not examine them. In general, an observation that expression of genes induced by TGF-β has been reduced by IFN-γ therapy will be more interpretable. In fibroblasts, up-regulation by TGF-β of gene expression and production of the corresponding functional gene products, such as CTGF, Col1A1, Col1A2, and fibronectin, contributes greatly to the occurrence of fibrosis (Varga et al, 1987; Ignotz et al, 1987; Igarishi et al, 1993; Krupsky et al, 1996; Lasky et al, 1998; Chen et al, 1999; Eickelberg et al, 1999; Zhang et al, 2000). Paradoxically, up-regulation of integrin subunits α5, β1, and β2; proteases including MMP-2, MMP-9, and urokinase plasminogen activator; and urokinase plasminogen activator receptor gene expression in macrophages by TGF-β may contribute to tissue destruction (Wahl et al, 1993; Falcone et al, 1995; Bauvois et al, 1996; Tajirian et al, 2000). Down-regulation of TGF-β-induced gene expression by subsequent treatment with IFN-γ has been demonstrated directly for Col1A1 and Col1A2 (Yuan et al, 1999; Ghosh et al, 2001) or suggested by functional correlations for the α5 and β2 integrin subunits (Bauvois et al, 1996). Of particular note, decreased expression of CTGF and TGF-β itself has been implicated in amelioration of IPF by IFN-γ therapy (Ziesche et al, 1999).

A key aspect of how IFN-γ antagonizes TGF-β is independent of direct effects on effector genes. Rather, evidence continues to mount that expression of TGF-β receptors and TGF-β itself is reduced due to cross-talk from IFN-γ that disrupts positive feedback in the TGF-β system (Wahl et al, 1990; Brandes et al, 1991; Bloom et al, 1996; Seder et al, 1998; Kavsak et al, 2000; Tredget et al, 2000; Blanchette et al, 2001). Thus, it is important not only to examine expression of the genes that effect fibrosis or macrophage function, but also to understand whether this fundamental interaction between IFN-γ and TGF-β occurs during IFN-γ therapy for TB.

**Proposed experiments.** To determine the molecular effects of IFN-γ on the fundamental TGF-β system, each component of that system will be assessed in BAL fluid or BAL cells obtained prior to, during and at the completion of treatment. This will include measuring secreted TGF-β, cell surface TGF-β receptors, DNA-binding activity of SMAD-containing complexes, abundance of SMAD proteins and mRNAs, and mRNA abundance of genes regulated by TGF-β. Only gene expression (mRNA abundance) will be examined in samples obtained by transbronchial biopsy. Details of the methods and the sample sets for these experiments are provided below.

Active and total TGF-β will measured in BAL fluid and in 24 h supernatants from BAL cells cultured *ex vivo* by performing a bioassay before and after heat treatment of the samples. Analysis of TGF-β receptors on total BAL cells or sorted AM (see Aim 2) will be performed by ligand binding. Activated SMAD-containing complexes in protein extracts from BAL cells will be measured by electrophoretic mobility shift assay (EMSA) with several SMAD-binding element (SBE) probes. The identity of the SMADs in the complexes will be determined by including specific antibodies in the EMSA reactions. The abundance of SMAD2, SMAD3, SMAD4, and SMAD7 proteins will be determined by immunoblot. SMAD mRNA abundance will be measured by quantitative real-time RT-PCR with molecular beacons.

A major molecular endpoint for these studies is to measure expression of genes that have previously been shown to be counter-regulated by TGF-β and IFN-γ or that are likely to be altered by the effect of IFN-γ on the TGF-β system itself. Gene expression will be assayed by measuring the mRNA abundance (as above) of TGF-β species, TGF-β receptors (type I, II, and III), Col1A1, Col1A2, CTGF, integrin subunits α5 and β2, MMP-2, and MMP-9. Given sufficient tissue sample, we will evaluate adhesion molecules such as ICAM and pCAM. These genes have been chosen as a representative subset of genes induced by TGF-β in fibroblasts and/or macrophages, including those already known to be counter-regulated by IFN-γ. Moreover, most of these have a clear or likely link to pathophysiology. It is known that human lung fibroblasts and AM express many of these genes normally, particularly TGF-β itself, and that several inflammatory disease states (silicosis, IPF, TB) are associated with increased expression of TGF-β and TGF-β-regulated genes (Broekelmann et al, 1991; Chang et al, 1996; Jagirdar et al, 1996; Lasky et al, 1998; Ziesche et al, 1999; Aung et al, 2000; Coker et al, 2001; Ramos et al, 2001). Inflammatory stimuli induce expression of TGF-β and TGF-β-regulated genes *ex vivo* (Dahl et al, 1996; Toossi et al, 1996; Hirsch et al, 1999), and cells from TB patients produce more TGF-β than cells from normal donors (Hirsch et al, 1999), although it has been found that AM from healthy donors produce less TGF-β than peripheral blood monocytes when both are stimulated with LPS (Toossi et al, 1996). TGF-β-induced expression of TGF-βR1 (Bloom et al, 1996), Col1A1 (Krupsky et al, 1996), and CTGF (Lasky et al, 1998) in human lung fibroblasts also has been demonstrated *ex vivo*. Examining the expression of these genes will provide important functional data about whether IFN-γ antagonism of the TGF-β system is a molecular basis for clinical improvement in response to IFN-γ therapy for TB.

**Expected results and interpretation.**

*Effects on TGF-β and TGF-β receptors:* Our hypothesis is that beneficial effects of IFN-γ will be due, at least in part, to antagonizing the TGF-β system. We expect to find that active TGF-β species and TGF-β receptors are reduced during this trial, which would be an important functional basis for antagonism. Reduced levels of active TGF-β species or TGF-β receptors also would be a significant indication that antagonism of the TGF-β system by aerosolized IFN-γ was responsible for other molecular changes and was a foundation for observed clinical effects. Reductions in TGF-β and TGF-β receptor gene expression are expected to occur as well. These changes in gene expression would underlie the changes in the level of TGF-β and TGF-β receptors and suggest that interference with positive feedback regulation in the TGF-β system was a basis for antagonism *in vivo*. We predict that aerosol rIFN-γ will have more significant effects in BAL cells whereas subcutaneous injection may lead to changes in gene expression on the tissue level.

*Effects on SMAD-containing DNA-binding activities:* Overall, reduction of SMAD-containing complexes that are detected by EMSA is expected as a consequence of reductions in active TGF-β and TGF-β receptor. Such results would suggest that activation of SMAD-2 and SMAD-3 and formation of their complexes with SMAD-4 had decreased, although confirmation of these mechanistic details is beyond the scope of this proposal. However, the DNA-binding activity detected by probes based on the SBEs from different genes may vary. Such differences will indicate whether decreased DNA-binding activity of SMAD-containing complexes or alternative mechanisms predominate for regulation of different genes in the various cell types present in biopsy and BAL samples. It is clear that SMAD-2 and SMAD-3 have different functions and do not necessarily regulate the same genes (Piek et al, 2001 and references therein). Thus, it will be important to determine which SMADs are in which complexes by performing reactions with antibodies. While a decrease in DNA-binding activity of SMAD-containing complexes, by whatever mechanism, should reduce downstream gene expression, TGF-β-regulated expression of some genes can also be inhibited without such a change.

*Effects on TGF-β-regulated gene expression and SMAD protein abundance:* Expression of most of genes positively regulated by TGF-β is expected to decrease; thus, that result should be seen among the genes that we plan to assay. However, the effect of IFN-γ on SMAD-7 gene expression is harder to predict because it is induced by both TGF-β and IFN-γ (Nakao et al, 1997; Ulloa et al, 1999). SMAD-7 is known to inhibit SMAD-2 and SMAD-3 activation (Hayashi et al, 1997; Nakao et al, 1997; Ulloa et al, 1999) and to promote TGF‑β receptor turnover (Kavsak et al, 2000). It is likely that SMAD-7 protein and mRNA will be readily detectable before treatment with IFN-γ. TGF-β-regulated expression of SMAD-7 should decrease due to antagonism of the TGF-β system by IFN-γ. Nonetheless, a net increase in SMAD-7 gene expression and protein abundance may contribute to a decrease in expression of genes that are negatively regulated by SMAD-7. Such a combination of changes in gene expression would be sufficient to confirm that IFN-γ functionally counteracts effects of endogenous TGF-β at the molecular level *in vivo*.

Little is known about interactions between TGF-β and IFN-γ in the regulation of SMAD-2, SMAD-3 and SMAD-4 gene expression. Thus, it is not possible to predict how IFN-γ will affect their mRNA or protein abundance. These assays will indicate whether changes in SMAD-containing DNA-binding complexes are due at least in part to regulation of SMAD protein abundance, either independent of or because of regulation of SMAD mRNA abundance. These data also will allow conclusions to be drawn about whether regulated SMAD gene expression contributes to the clinical effects of treatment by IFN-γ.

Taken together, these data on changes in components of the TGF-β system and counter-regulated gene expression will provide molecular links between treatment of patients with IFN-γ and beneficial clinical effects. They will also provide a biomedical foundation for future basic research on the interaction between IFN-γ and TGF-β in model systems and for consideration of more targeted clinical intervention in the TGF-β system.

**Methods.**

*Measurement of TGF-β and TGF-β receptors:* Total and active TGF-β will be measured in cell culture media conditioned by growth of BAL cells and in BAL fluid concentrated and desalted by centrifugal ultrafiltration. A TGF-β bioassay has been developed by our group (Munger et al, 1999), and Dr. Munger will serve as a consultant for these assays (see attached letter). Mink lung epithelial cells have been stably transfected with a plasmid containing the luciferase cDNA downstream of a TGF-β-sensitive portion of the plasminogen activator inhibitor 1 promoter. Cells are suspended in DMEM/10% FCS at 5 x 10^5^ cells/ml and plated at 50 μl per microtiter well for 1 hr to adhere. Medium is replaced to 50 μl/well of the same medium plus 50 μl of test solution and cultured for 16-20 hr. Lysates are then assayed for luciferase activity. This bioassay is sensitive and specific, and can be used to measure not only active TGF-β, but also total TGF-β. Any latent TGF-β present in the test solution will be activated by heating at 80° for 10 minutes prior to starting the assay. Unlike acidification to activate latent TGF-β, this procedure is compatible with the bioassay. The difference between the active TGF-β present before and after heating corresponds to the latent TGF-β that was present in the sample. The absolute concentrations of TGF-β in the test samples can be determined by comparison to a standard curve generated by assaying recombinant TGF-β. The specificity of each assay can be confirmed by including the 1D11 pan-neutralizing anti-TGF-β monoclonal antibody in a parallel control assay.

Cell surface TGF-β receptors are most often measured by cross-linking of [^125^I]-TGF-β. We will perform this assay essentially as described (Yuan et al, 1999). Radiolabeled ligand (3000-4500 Ci/mmol, New England Nuclear) will be reacted with BAL cells or isolated AM in the presence or absence of excess unlabeled TGF-β, then cross-linked with disuccinimidyl suberate. After extensive washing, cells are lysed and lysates are analyzed by SDS-PAGE. The receptors are identified by the specificity of their labeling and by their electrophoretic mobility, and can be quantified either by autoradiography and densitometry, or by directly measuring radioactivity in bands cut from the gel. This assay is sensitive enough to detect as few as 100 receptor complexes per cell in a sample of 10^6^ cells, assuming equal labeling of each receptor subunit. With as little as 50% labeling efficiency, each receptor subunit would be tagged with approximately 200 dpm, which is readily detectable by autoradiography. Expert assistance in performing this assay (and measuring DNA-binding activity of SMAD-containing complexes) is available through collaboration with John Varga (see attached letter).

*Measurement of SMAD-containing DNA-binding activities and SMAD proteins:* The DNA-binding activity of SMAD-containing complexes and the abundance of SMAD proteins in whole cell extracts will be measured by EMSA and by immunoblot, respectively, essentially as described (Chen et al, 1999). We have previously measured STAT-1, IRF-1, and IRF-9 in whole cell extracts of BAL cells (Condos et al, 2001). Recently, we have determined that EMSA with either of two different SBE probes shows little or no difference between SMAD-DNA binding activity recovered in whole cell extracts or in nuclear extracts of TGF-β-treated THP-1 cells (R. Pine, unpublished).

Extracts from patient BAL cells are prepared at 4° in a BSL-3 laboratory. All centrifugation is performed in Porton-Down certified aerosol containment rotors, and all manipulations are performed inside a laminar flow hood. Preparing extracts entails only washing cells in phosphate buffered saline, incubating them in extraction buffer for 30 min at 4°, removing debris by centrifugation (13,000 x g, 15 minutes) and recovering the supernatant. The extracts are sterilely filtered before being removed from the BSL-3 laboratory for later assay. Extracts are stored at –80°. The extraction buffer consists of 0.5% nonidet P40, 10% glycerol, 0.1 mM EDTA, 20 mM NaCl, 0.3 M NaCl, and freshly added DTT (1 mM), protease inhibitors (3 µg aprotinin, 1 µg leupeptin, and 2 µg pepstatin per ml, and 2 mM PMSF), and phosphatase inhibitors (30 mM Na_4_P_2_O_7_, 1 mM NaF, and 10 µM Na_3_VO_4_). For EMSA, extracts (3 µg total protein) are incubated with radiolabeled oligonucleotide probe (0.3 - 0.5 ng, 4 - 5 X 10^4^ dpm) in buffer that contains 1 mM MgCl2, 1.0 mM DTT, 20 mM Hepes, pH 7.9, 0.1 mM EGTA, 0.1 mM EDTA, 160 µg poly(dIdC:dIdC) per ml, 0.2 % nonidet P40, 3% glycerol and 4% ficoll for 30 minutes at room temperature. Oligonucleotide competitors (100 fold molar excess) or antibodies (1-2 µg per reaction) are included throughout the incubation when used. Samples are electrophoresed on 6% polyacrylamide gels with 20 mM Tris-borate, pH 8.3, 2 mM EDTA buffer at 4°. We obtain images and quantify the results of EMSA with a phosphorimager (Storm 860, Molecular Dynamics). In addition to the use of a consensus SMAD-binding element (SBE) (Zawel et al, 1998) as a standard for detection of SMAD-containing complexes, we will perform assays with the SBEs from the Col1A2 (Chen et al, 1999), CTGF (Holmes et al, 2001), and SMAD-7 (von Gersdorff et al, 2000) promoters to determine changes in SMAD-containing complexes that act on those genes. Appropriate controls with competitor oligonucleotides and antibodies will be performed to definitively identify the complexes detected.

Immunoblots to detect SMADs will be performed with standard procedures in use in the laboratory and commercially available antibodies (Zymed Laboratories or Santa-Cruz Biotechnolgy). Samples separated by SDS-PAGE are electro-transferred to nitrocellulose or polyvinylidene difluoride membranes, as appropriate. The membranes are blocked in a solution of 5% nonfat dry milk and 0.2% Tween-20 in Tris-buffered saline prior to incubation with primary antibody. The membranes are washed and then incubated with horseradish peroxidase conjugated, species-specific secondary antibody to detect the specifically bound primary antibody. The membranes are washed again and then developed with either enhanced chemiluminescence for high sensitivity, semi-quantitative detection or, when there is sufficient material, enhanced chemifluoresence for quantitative measurement. The STORM 860 also has a capability for quantitative two-dimensional analysis of fluorescent samples. For each BAL cell sample, approximately 250 µg of protein can be obtained from 5 x 10^6^ cells. That is enough material for 24 individual EMSAs (with nonspecific or specific competitor oligonucleotide and with nonspecific or specific antibodies, for each of the 4 probes) and for immunoblots (30 µg protein per lane)

*General strategy (molecular characteristics for correlation with clinical results):* The overall approach for part **a** of this aim is to determine whether known molecular mechanisms by which IFN-γ antagonizes effects of TGF-β operate *in vivo* in the lung during treatment with aerosolized IFN-γ in ways that correlate with and might explain clinical effects. We can achieve our goal for part **a** of this aim by determining at a minimum whether there are changes in levels of TGF-β and corresponding changes in TGF-β-regulated gene expression. Thus, it is not strictly necessary to determine how changes in signal transduction and transcriptional regulation, or in the expression of the molecules that effect these processes, mediate the outcome. Nonetheless, the experiments to examine molecular mechanisms that operate *in vivo* in the lung to antagonize effects of endogenous TGF-β during treatment with aerosolized IFN-γ are well warranted. Whether we obtain expected or unexpected results, the data will provide important details for rationalization of this therapy or for development of new, even more specific interventions.

The expected results we have described are based on data from studies in many different systems, but have been colored most by limited studies of human lung samples, including tissue biopsies or isolated fibroblasts and bronchoalveolar lavage cells (Broekelmann et al, 1991; Krupsky et al, 1996; Lasky et al, 1998; Ziesche et al, 1999; Eickelberg et al, 1999; Aung et al, 2000; Ramos et al, 2001; Coker et al, 2001). While most of the anticipated changes in gene expression are predicted confidently, the details of intermediate steps may be other than those expected. Much has been learned recently about the remarkable combinatorial complexity that apparently determines cell type and context specific activation or inhibition of gene expression in the TGF-β system (reviewed in Piek et al, 1999; Roberts, 1999; Itoh, 2000; Massague & Chen, 2000; Massague & Wotton, 2000; Zimmerman & Padgett, 2000; Wotton & Massague, 2001). Surprisingly, the specific basis for regulation of particular genes in particular circumstances is not well documented. For example, the mechanisms that mediate a key aspect of antagonistic cross-talk between IFN-γ and TGF-β (down-regulation of TGF-β and TGF-β receptor expression at the protein and RNA level in response to IFN-γ) are not well understood. We have described interpretation of the expected results in terms of known underlying mechanisms that could account for them. Unexpected data on signal transduction in the TGF-β system and antagonism by IFN-γ would necessarily suggest alternative mechanisms. It will not be feasible to confirm the possible alternatives in studies of clinical samples. Rather, such results will provide the impetus for additional research with model systems that are more amenable to experimental manipulation and control. The data also would serve to validate whether a model system reflects the *in vivo* situation and to provide a foundation for future mechanistic studies in such a model system.

It must also be noted that the interaction between the TGF-β system and IFN-γ can have cell-type specific effects that lead to quite different changes in gene expression mediated by different mechanisms. However, planning to study expression of genes in BAL cells and biopsy specimens (primarily fibroblasts, myofibroblasts, and epithelial cells) that are regulated in a predominantly cell-type specific manner addresses this concern. This approach will also be robust enough to demonstrate effects of therapy on the TGF-β system in BAL cells despite changes in cellularity that typically occur during the course of clinical improvement. TGF-β effects on T cells have been studied in various contexts, including that of TB (Toossi & Ellner, 1998 and references therein; Hirsch et al, 1999; Othieno et al, 1999; Rojas et al, 1999; Zhang et al, 1999; Ludviksson et al, 2000). However, alveolar macrophages predominate in BAL cells even when significant alveolitis is present. Similarly, although therapy that affects the TGF-β system might alter cell proliferation and thus the proportion of fibroblasts, myofibroblasts and epithelial cells in a biopsy sample, changes in cell-type specific gene expression should still be evident. Nonetheless, any molecular biological data we obtain that do reflect cellular changes will reveal functional correlates of those changes.

The PI of the grant will supervise division of cells from BAL sampling to achieve the goals of the grant with the general rule that half will be directed toward Aim 2 and half to Aim 3 (~20-25 x 10^6^ cells for each aim out of a typical 40-50 x 10^6^ BAL cell lavage). Thus, it is expected that the amount of TGFβ in BAL fluid or after *ex vivo* culture of BAL cells, the cell surface TGFβ receptor abundance, the DNA-binding activity of SMAD-containing complexes, and levels of gene expression will be measured for all BAL samples. Levels of gene expression also will be measured for all biopsy samples. For each parameter, we will calculate means, variance, and confidence intervals for comparison of patients being treated with placebo or IFNγ. This will allow us to determine whether there are statistically significant differences between pre-treatment and treatment values within each group of patients and between values obtained for placebo and IFNγ groups at different times of treatment. Differences of more than two-fold will be accepted as statistically significant for P values of < 0.05. Differences between 1.5 and 2.0 fold will be considered significant for P values of < 0.01. Smaller differences will be scored only for P values of < 0.005. Even if differences are not statistically significant, we expect the data from individual patients to demonstrate consistent trends in the directions described in the expected results.

*Preparation of extracts (RNA and protein samples):* Protein extracts are very stable as long as they are kept frozen below -60°. Similarly, RNA prepared from biopsy specimens or BAL cells using the RNeasy kit is very pure and remains intact for prolonged periods when kept frozen below –60°. Thus, RNA and protein extracts prepared by our collaborators at the University of Cape Town in South Africa can be shipped here on dry-ice and will arrive in excellent condition.

*Measurement of TGF-β species and TGF-β receptors:* Despite the range of cell types that might have secreted TGF-β recovered in BAL fluid or in a culture of BAL cells *ex vivo*, it is likely that TGF-β1 will predominate. Although addition of subtype-specific neutralizing antibodies to the bioassay can allow determination of the different TGF-β subtypes that might be present, this approach relies on careful controls to avoid ambiguity arising from antibody cross reactivity. Furthermore, it would be quite difficult to reliably quantify minor constituents. As little information is likely to be gained relative to the extra effort required, we have planned only to use a pan-reactive antibody as a control for the specificity of TGF-β measurement. We presently plan to assay TGF-β receptors only on the surface of BAL cells. Our goal is to determine if down-regulation of cell-surface receptors might contribute to antagonism of the TGF-β system by aerosolized IFN-γ.

## *Gene expression (molecular beacon assays):* We have already successfully performed molecular beacon assays for several genes in this laboratory (Y. Qiao and R. Pine, unpublished). We routinely obtain RNA free of DNA contamination from BAL cells or THP-1 cells using the protocol described in aim 2. These experiments have confirmed induction of specific genes during infection by *Mtb t*hat was first detected by functional genomic assays. As well, we have used this method to study regulation of IRF-1 and IP-10 gene expression in the context of infection by *Mtb* and response to IFN-α or IFN-γ. The primer and beacon set for glyceraldehyde phosphate dehydrogenase, a housekeeping gene that mediates a key step in glycolysis, also is in use in the laboratory in conjunction with these ongoing studies. While this experience has confirmed that we can design oligonucleotides that usually work well under the intended conditions, optimization may be required to obtain sufficient specificity or sensitivity. We have successfully optimized conditions for oligonucleotide sets that did not work as designed. Thus, we anticipate little or no difficulty in obtaining the desired data on gene expression. However, informal consultations with Sanjay Tyagi and Fred Kramer, colleagues at PHRI who invented molecular beacon technology, will help to overcome any difficulty in design and use of primer-beacon sets that might arise for one or another target.

**E. HUMAN SUBJECTS.**

This project will be conducted at NYU/Bellevue Hospital in New York City and Groote Schuur Hospital at the University of Cape Town. The clinical protocol described in this grant and an identical informed consent will be submitted to the IBRA at both locations. The reviews at the NYU School of Medicine IBRA also include a Research Review Committee at Bellevue Hospital and the Health and Hospitals Corporation as well as the scientific review of the Executive Advisory Committee of the NYU General Clinical Research Center (GCRC). The same strict standards will be applied from each and every review committee.

**1. Risks to the Subjects**

**Human subjects involvement and characteristics:** Medical evaluations will be performed on 40 individuals with active bilateral, cavitary pulmonary TB over 5 years. All patients with TB will be randomized to receive IFN-γ subcutaneous (n=32) plus DOTS treatment. Patients must meet inclusion/exclusion criteria outlined under Aim 1 in the Research Design and Methods section above. There must be no contraindication to fiberoptic bronchoscopy. The study population will likely be over 60% minorities, aged 18-75, and have a 4:1 male:female ratio, reflecting the population of TB-infected individuals. Patients with HIV-1 infection will be stratified in both groups. Only patients not previously receiving antiretroviral therapy, particularly with protease inhibitors, will be enrolled, and protease inhibitors will be held during the first four months of TB therapy. This is in accordance with recent CDC guidelines for use of protease inhibitors in AIDS patients with TB and the Virology Service at Bellevue Hospital, where the TB treatment is completed before HAART therapy is initiated.

**Sources of materials:** Research materials to be obtained from the subjects include blood, urine and sputum samples, pregnancy test for female subjects, EKG, chest radiograph and chest CT scans. We will be performing research tests on PBMC, time to culture *Mtb* in sputum, and clinical response to rIFN-γ with CT scans. Each patient will have three fiberoptic bronchoscopies with bronchoalveolar lavage (BAL), and BAL cells and fluid will be used for research. On the first and third bronchoscopy, the patients will also undergo a transbronchial lung biopsy to obtain a research specimen. This specimen will be used to analyze genes/proteins related to lung fibrosis/destruction and the effect of standard antituberculous treatment compared to standard treatment plus 4 months rIFN-γ.

**Potential risks:** In general, the risk and severity of side effects to interferon-gamma (IFN-γ) are related to the amount of medication given. At the dose used in this study (200 mcg), most common possible side effects of rIFN-γ include fever, headache and malaise. Occasional nausea and vomiting have been reported at high doses. Aerosolization has not been associated with adverse reactions, although headache, cough and fever may be expected. In the event of severe symptoms, medication will be stopped.

Risk from bronchoalveolar lavage (BAL) is primarily post-procedure fever which is transitory and occurs in 5% of patients and <1% of normals. Fever is probably from bacteria introduced into the lower respiratory tract from the bronchoscope passing through the upper respiratory tract. The transitory fever is treated with acetaminophen. Pneumonia is a rare complication. Complications of BAL and transbronchial biopsy include bleeding or pneumothorax, although these are rare. Clotting parameters must be normal prior to BAL and a chest x-ray is done to rule out pneumothorax post-procedure. Epinephrine 1:20,000 is instilled in the airway to stop bleeding after transbronchial biopsy, and suction is applied with the bronchoscope in the wedge position. There is risk from lidocaine 1% in the local anesthesia of vocal cords, trachea and airways, where excess lidocaine can induce seizures, and even death. To avoid this, limits on lidocaine use have been instituted and described in the informed consent. Transbronchial lung biopsies are necessary to study the TGF-β gene profile; the rIFN-γ treatment provides a unique opportunity to obtain these measurements.

There may be transitory pain associated with venipuncture.

**2. Adequacy of Protection against Risks**

**Recruitment and Informed Consent:** Subjects with active TB will be recruited from the Bellevue Chest Service and Groote Schuur Hospital and clinics of the University of Cape Town. Recruitment will be done by pulmonary faculty, who will discuss the project with prospective study subjects and obtain informed consent with a nurse witness. Individuals will be selected without regard to sex, race or ethnicity. The informed consent forms are under review by the NYU and University of Cape Town respective IBRAs.

**Protection against Risk:** To minimize any risks, BAL and transbronchial biopsy are performed after medical evaluation excluding individuals with cardiac disease or history of angina. Chest x-ray and blood studies including bleeding parameters (platelets, prothrombin time, PTT) are performed. BAL will be performed by pulmonary fellows under faculty supervision. The transbronchial lung biopsy will be performed by the pulmonary faculty, and the bronchoscope will be maintained in the wedge position with suction for any bleeding. At the bronchoscopist’s discretion, epinephrine (1:20,000) is instilled, if necessary, to stop bleeding.

Following the procedure, the study subjects will remain NPO for 3 hr and vital signs will be taken every 30 min for 3 hr. All patients will have their EKG monitored during the procedure and will receive nasal O_2_ during and after the procedure for 2 hr to prevent any hypoxia. All patient data will be kept locked in the pulmonary research offices. In the event of adverse effects to subjects, a “crash cart” is kept with the fiberoptic bronchoscope, including endotracheal tubes, injectable lidocaine and epinephrine, etc, and all procedures are done in the hospital with house staff and a CPR team on call. All of the BAL and transbronchial biopsy procedures will be periodically reviewed to identify any increased incidence of untoward effects and identify their cause.

To monitor risks, the NYU GCRC has hired a Research Subjects Advocate (Andrea Downey, RN, MS), who will review all clinical protocols, informed consents, and IBRA submissions. She will audit the informed consent process as well as all of the clinical data and the bronchoscopy records at both sites. She reports directly to the Dean of the NYU School of Medicine. See Data and Safety Monitoring Plan below.

**3. Potential Benefits of the Proposed Research to the Subjects and Others**

The risks to subjects in this study are minimal and acceptable in comparison to the benefits to be accrued in studying cells obtained from the site of disease in the lower respiratory tract. This is the only way to study the fundamental mechanisms of TB in humans. Knowledge about the function of these cells can be gained at the protein and gene level.

**4. Importance of the Knowledge to be Gained**

We propose a randomized, controlled clinical trial to test a promising new therapy of rIFN-γ in the most difficult drug-sensitive TB to treat: AFB smear positive with bilateral disease and one or more cavities. This is an efficacy clinical trial that, if rIFN-γ works, may pioneer a new treatment modality.

**Inclusion of Women:** Women will be included in this study. They need to have a negative pregnancy test within 48 hours of starting this study. The sex predominance of our patients with TB is 4:1 male:female and our study population will be the same.

**Inclusion of Minorities:** Minorities will be included in this study. Our TB patients are 60% Black and Hispanic. The age range is predominantly 15-44 yr. Most of the patients recruited in South Africa will be Black or South Asian (India). The following table demonstrates the ethnic and racial distribution of our subjects:

# Inclusion Enrollment Report Table

| **Study Title:** Subcutaneous Interferon –γ for TB/HIV | | | | | | |
| --- | --- | --- | --- | --- | --- | --- |
| **Total Enrollment:** | | Protocol Number: H | | | | |
| Grant Number: | |  | | | | |
|  | | | | | | |
| **PART A. TOTAL ENROLLMENT REPORT: Number of Subjects Enrolled to Date (Cumulative)**  **by Ethnicity and Race** | | | | | | |
| Ethnic Category | **Sex/Gender** | | | | | |
|  | **Females** | | **Males** | **Unknown or Not Reported** | **Total** | |
| Hispanic or Latino |  | |  |  |  | ** |
| Not Hispanic or Latino |  | |  |  |  |  |
| Unknown (Individuals not reporting ethnicity) | 0 | | 0 | 0 |  |  |
| Ethnic Category: Total of All Subjects* |  | |  | 0 |  | * |
| Racial Categories |  | |  | | | |
| American Indian/Alaska Native | 0 | | 0 | 0 | 0 |  |
| Asian |  | |  | 0 |  |  |
| Native Hawaiian or Other Pacific Islander | 0 | | 0 | 0 | 0 |  |
| Black or African American |  | |  | 0 |  |  |
| White |  | |  | 0 |  |  |
| More than one race | 0 | | 0 | 0 | 0 |  |
| Unknown or not reported | 0 | | 0 | 0 | 0 |  |
| **Racial Categories: Total of All Subjects*** |  | |  | 0 |  | * |

|  | | | | | |
| --- | --- | --- | --- | --- | --- |
| PART B. HISPANIC ENROLLMENT REPORT: Number of Hispanics or Latinos Enrolled to Date (Cumulative) | | | | | |
| **Racial Categories** | **Females** | **Males** | **Unknown or Not Reported** | Total | |
| American Indian or Alaska Native |  |  |  |  |  |
| Asian |  |  |  |  |  |
| Native Hawaiian or Other Pacific Islander |  |  |  |  |  |
| Black or African American |  |  |  |  |  |
| White |  |  |  |  |  |
| More Than One Race |  |  |  |  |  |
| Unknown or not reported |  |  |  |  |  |
| **Racial Categories: Total of Hispanics or Latinos**** |  |  |  |  | ** |
| * These totals must agree.  ** These totals must agree. |  |  | | | |

**Inclusion of Children:** Children will not be included in this study, because insufficient data are available from adults to judge potential risk in children. Fiberoptic bronchoscopy is more difficult in children and requires a pediatric bronchoscope. Children usually have primary TB, which presents differently from reactivation TB in adults.

**Data and Safety Monitoring Plan** is to provide for safety of study subjects and validity/integrity of the data. The study is designed to test the hypothesis that aerosol IFNγ over 4 months will improve mycobacterial response, HRCT resolution, BAL/PBMC cellular and inflammatory response, and lessen fibrotic stimuli as demonstrated by the TGFβ/SMAD pathway in bilateral, cavitary pulmonary TB patients.

A DSM Board has been established with David Prezant, MD, Professor of Pulmonary Medicine at Albert Einstein College of Medicine, as Chair. He has expertise in bronchoscopy and clinical trials research. Members include Jerrold Ellner, MD, Chairman of Medicine at UMDNJ-Newark and Gilla Kaplan, PhD at the PHRI at UMDNJ-Newark. They are international authorities on TB and have extensive experience in conducting clinical trials in Africa. Philip Alcabes, PhD, Associate Professor, Hunter College at CUNY, is our biostatistician. He has > 5 years of clinical trials experience as a GCRC biostatistician. Dr. Elvis Irusen has joined our DSMB. He is a Professor of Medicine and Head of the Pulmonary Division at the University of Stellenbosch and Tygerberg Hospital.

The DSMB will meet every 6 months to review each research subject’s progress. The DSMB will keep minutes of their meetings and provide these to the relevant NIH Program officer. The DSMB will review unblinded data on endpoints: CT scans, smears/cultures, BAL/PBMC flow assays, and basic science studies. Drs. Rom and Ress will be involved in patient care, but they will be blinded as to all of the study results.

While we propose that the Data Safety Monitoring Committee develop a plan for the monitoring of safety and efficacy in these patients based on a series of assessments including a plan for terminating the trial early in the event of extreme interim results, we are including here one sample interim monitoring plan for a combined safety/efficacy endpoint.

The DSMB will be notified of all serious adverse events immediately. They will review the data, the circumstances of the event and in consultation with Drs. Rom and Ress, require changes in the protocol or procedures. The DSMB will function as an advisor, but also as a regulatory body with line function as well as a staff advisory role. The monitoring plan will review the clinical data, medication record, bronchoscopy procedures, and scientific data accumulation. The first function of the DSMB is to monitor the clinical study. They need to evaluate accrual, selection of patients, inclusion/exclusion criteria, informed consent, treatment protocol, and bronchoscopy data. The second function is to review all bronchoscopy records since this is the high risk portion of the research. Third, the DSMB must review data quality including BAL cells (Quantity), transbronchial biopsies (enough material), CT-scans (quality, technique), and flow cytometry data. Fourth, the DSMB must monitor the science, especially the molecular biology of the TGF-β system. Fifth, the DSMB must monitor clinical endpoints (e.g. cultures, smears, symptoms, change in CT scan cavities, etc.). Sixth, the DSMB needs to evaluate interaction between the various sites. Seventh, the DSMB will monitor quality control and performance, especially adherence to the protocol at both sites.

Sample Interim Monitoring Plan:

If we have an interest in rejecting the null hypothesis that there is no difference between the two treatment groups with respect to the median time to a treatment failure or positive smear present with two samples of size 32, a sample interim monitoring plan is proposed.

If we assume a 50% failure rate for the standard treatment at 6 months and a 50% failure rate for the new treatment at 18 months, with 2 years of accrual and a four year study, safety monitoring would reject the null hypothesis with an overall 2- sided alpha level of 0.05 and power of 80% (O-Brien –Fleming boundaries) with 3 interim looks and one final look (one look/year) as shown below:

The DSMB will evaluate proposed stopping rules and develop milestones for accrual at their first meeting. During the 03 year, we may accrue sufficient subjects and samples for an interim analysis.

**Individual Stopping Rules.** The DSMB will establish individual stopping rules. For example, adverse events of grade 3 or 4 common toxicity criteria will warrant removal from the clinical trial (Appendix 1). Specific criteria will include significant exacerbation on the chest x-ray/CT scan as judged by the study radiologist (David Naidich, MD) and/or in those with HIV/Mtb co-infection, decline in CD4+ cells < 50/μl and/or increase in plasma HIV-1 viral load > 10^5^ copies/μl at months 1, 2, or 3.

Patients with bacteriologic failure defined as persistent positive AFB smears at 5 months (ATS statement 1994) will be removed from the clinical trial for clinical evaluation.

**F. VERTEBRATE ANIMALS.** Not applicable.

## G. LITERATURE CITED

Aber, V.R. and Nunn, A.J. 1978. Factors affecting relapse following short-course chemotherapy. *Bull Int Union Against Tuberculosis* 53:260-264.

Adema, G.J., Hargers, F., Verstraten, R., de Vries, E., Marland, G., Menon, S., Foster, J., Xu, Y., Nooyen, P., McClanahan, T., Bacon, K.B., Figdor, C.G. 1997. A dendritic-cell-derived C-C chemokine that preferentially attracts naïve T cells. *Nature* 387:713-717.

Albelda, S.M. and Sheppard, D. 2000. Functional genomics and expression profiling: be there or be square. *Am J Respir Cell Mol Biol*  23:265-269.

Aung, H., Toossi, Z., Mckenna, S.M., Gogate, P., Sierra, J., Sada, E., Rich, E.A. 2000. Expression of transforming growth factor-beta but not tumor necrosis factor-alpha, interferon-gamma, and interleukin-4 in granulomatous lung lesions in tuberculosis. *Tuber Lung Dis*  80:61-67.

Barnes, P.F. and Barrows, S.A. 1993. Tuberculosis in the 1990’s. *Ann Intern Med* 119:400-410.

Bass, J.B.J., Farer, L.S., Hopewell, P.C., et al. 1994. Treatment of tuberculosis and tuberculosis infection in adults and children. *Am J Respir Crit Care Med* 149:1359-1374.

Bauvois, B., Van Weyenbergh, J., Rouillard, D., Wietzerbin, J. 1996. TGF-beta 1-stimulated adhesion of human mononuclear phagocytes to fibronectin and laminin is abolished by IFN-gamma: dependence on alpha 5 beta 1 and beta 2 integrins. *Exp Cell Res* 222:209-217.

Blanchette, F., Rudd, P., Grondin, F., Attisano, L., Dubois, C.M. 2001. Involvement of Smads in TGFbeta1-induced furin (fur) transcription. *J Cell Physiol*  188:264-273.

Bloom, B.B., Humphries, D.E., Kuang, P.P., Fine, A., Goldstein, R.H. 1996. Structure and expression of the promoter for the R4/ALK5 human type I transforming growth factor-beta receptor: regulation by TGF-beta. *Biochim Biophys Acta*  1312:243-248.

Bluyssen, H.A.R., Durbin, J.E., Levy, D.E. 1996. ISGF3γ p48, a specificity switch for interferon activated transcription factors. *Cytokine Growth Factor Rev* 7:11-17.

Brandes, M.E., Wakefield, L.M., Wahl, S.M. 1991. Modulation of monocyte type I transforming growth factor-beta receptors by inflammatory stimuli. *J Biol Chem*  266:19697-19703.

Broekelmann, T.J., Limper, A.H., Colby, T.V., McDonald, J.A. 1991. Transforming growth factor beta 1 is present at sites of extracellular matrix gene expression in human pulmonary fibrosis. *Proc Natl Acad Sci USA* 188:6642-6646.

Cario, E., Rosenberg, I.M., Brandwein, S.L., Beck, P.L., Reinecker, H.C., Podolsky, D.K. 2000. Lipopolysaccharide activates distinct signaling pathways in intestinal epithelial cell lines expressing Toll-like receptors. *J Immunol* 164:966-972.

Chang, J.C., Wysocki, A., Tchou-Wong, K.-M., Moskowitz, N., Zhang, Y., Rom, W.N. 1996. Effect of *Mycobacterium tuberculosis* and its components on macrophages and the release of matrix metalloproteinases. *Thorax* 51:306-311.

Chen, S.J., Yuan, W., Mori, Y., Levenson, A., Trojanowska, M., Varga, J. 1999. Stimulation of type I collagen transcription in human skin fibroblasts by TGF-beta: involvement of Smad 3. *J Invest Dermatol*  112:49-57.

Cohen, P., Bouaboula, M., Bellis, M., Baron, V., Jbilo, O., Poinot-Chazel, C., Galiegue, S., Hadibi, E.H., Casellas, P. 2000. Monitoring cellular responses to Listeria monocytogenes with oligonucleotide arrays. *J Biol Chem*  275:11181-11190.

Cohn, D.L., Catlin, B.J., Peterson, K.L., et al. 1990. A b2-dose 6-month therapy for pulmonary and extrapulmonary tuberculosis: a twice weekly, directly-observed, and cost-effective regimen. Ann Intern Med 112:407-415.

Coker, R.K., Laurent, G.J., Jeffery, P.K., Du Bois, R.M., Black, C.M., Mcanulty, R.J. 2001. Localisation of transforming growth factor beta1 and beta3 mRNA transcripts in normal and fibrotic human lung. *Thorax*  56:549-56.

Condos R, Hull FP, Schluger NW, Rom WN, and Smaldone GC. 2003. Regional

deposition of aerosolized interferon-γ in pulmonary tuberculosis. *Chest,* 2004;125: 2146-55.

Condos, R., Raju, B., Canova, A., Zhao, B.-Y., Weiden, M., Rom, W.N., Pine, R. 2003. Recombinant interferon-gamma aerosol stimulates signal transduction and induces gene expression while suppressing HIV-1 in tuberculosis patients. *Infect Immun*. 71: 2058-2064

Condos, R., Rom, W.N., Liu, Y.M., Schluger, N.W. 1998. Local immune responses correlate with presentation and outcome in tuberculosis. *Am J Respir Crit Care Med* 157:729-735.

Condos, R., Rom, W.N., Schluger, N.W. 1997. Treatment of multidrug-resistant pulmonary tuberculosis with interferon-γ via aerosol. *Lancet* 349:1513-1515.

Cooper, A.M., Dalton, D.K., Stewart, T.A., Griffin, J.P., Russell, D.G., Orme, I.M. 1993. Disseminated tuberculosis in interferon-γ gene-disrupted mice. *J Exp Med* 178:2243-2247.

Dahl, K.E., Shiratsuchi, H., Hamilton, B.D., Ellner, J.J., Toossi, Z. 1996. Selective induction of transforming growth factor beta in human monocytes by lipoarabinomannan of Mycobacterium tuberculosis. *Infect Immun*  64:399-405.

Darnell, J.E., Jr. 1997. STATs and gene regulation. *Science* 277:1630-1635.

De Fougerolles, A.R., Chi-Rosso, G., Bajardi, A., Gotwals, P., Green, C.D., Koteliansky, V.E. 2000. Global expression analysis of extracellular matrix-integrin interactions in monocytes. *Immunity*  13:749-58.

Der, S., Zhou, A., Williams, B., Silverman, R.. 1998. Identification of genes differentially regulated by interferon α, β, or γ using oligonucleotide arrays. *Proc Natl Acad Sci USA* 95:15623-15628.

Durbin, J.E., Hackenmiller, R., Simon, M.C., Levy, D.E. 1996. Targeted disruption of the mouse Stat 1 gene results in compromised innate immunity to viral disease. *Cell* 84:443-450.

Dye, C., Scheele, S., Dolin, P., Pathania, V., Raviglione, M.C. 1999. Global burden of tuberculosis: Estimated incidence, prevalence, and mortality by country. *JAMA* 282:677-686.

Eickelberg, O., Kohler, E., Reichenberger, F., Bertschin, S., Woodtli, T., Erne, P., Perruchoud, A.P., Roth, M. 1999. Extracellular matrix deposition by primary human lung fibroblasts in response to TGF-beta1 and TGF-beta3. *Am J Physiol*  276:L814-24.

Epstein, M.D., Schluger, N.W., Davidow, A.L., Bonk, S.B., Rom, W.N., Hanna, B. 1998. Time to detection of *Mycobacterium tuberculosis* in sputum culture correlates with outcome in patients receiving treatment for pulmonary tuberculosis. *Chest* 113:379-386.

Falcone, D.J., Mccaffrey, T.A., Mathew, J., Mcadam, K., Borth, W. 1995. THP-1 macrophage membrane-bound plasmin activity is up-regulated by transforming growth factor-beta 1 via increased expression of urokinase and the urokinase receptor. *J Cell Physiol*  164:334-343.

Fenton, M.J., Vermeulen, M.W., Kim, S., Burdick, M., Strieter, R.M., Kornfeld, H. 1997. Induction of gamma interferon production in human alveolar macrophages by *Mycobacterium tuberculosis*. *Infect Immun* 65:5149-5156.

Flodstrom, M, and Eizirik, D.L. 1997. Interferon-γ-induced interferon regulatory factor-1 (IRF-1) expression in rodent and human islet cells precedes nitric oxide production. *Endocrinology* 138:2747-2753.

Flynn, J.L., Chan, J., Triebold, K.J., Dalton, D.K., Stewart, T.A., Bloom, B.R. 1993. An essential role for interferon-γ in resistance to *Mycobacterium tuberculosis* infection. *J* *Exp Med* 178:2249-2254.

Friedman, C., Quinn, G., Kreiswirth, B., Perlman, D., Salomon, N., Schluger, N., Lutfey, M., Berger, J., Poltoratskaia, N., Riley, L. 1997. *J Infect Dis* 176:478-484.

Ghosh, A.K., Yuan, W., Mori, Y., Chen, S., Varga, J. 2001. Antagonistic regulation of type I collagen gene expression by interferon-gamma and transforming growth factor-beta. Integration at the level of p300/CBP transcriptional coactivators. *J Biol Chem*  276:11041-11048.

Giosue, S., Casarini, M., Ameglio, F., Zangrilli, P., Palla, M., Altieri, A.M., Bisetti. A. 2000. Aerosolized interferon-alpha treatment in patients with multi-drug-resistant pulmonary tuberculosis. *Eur Cytokine Netw* 11:99-104.

Golub, T.R., Slonim, D.K., Tamayo, P., Huard, C., Gaasenbeek, M., Mesirov, J.P., Coller, H., Loh, M.L., Downing, J.R., Caligiuri, M.A., Bloomfield, C.D., Lander, E.S. 1999. Molecular classification of cancer: class discovery and class prediction by gene expression monitoring. *Science*  286:531-537.

Gurujeyalakshmi, G., Giri, S.N. 1995. Molecular mechanisms of antifibrotic effect of interferon gamma in bleomycin-mouse model of lung fibrosis: downregulation of TGF-β and procollagen I and III gene expression. *Exp Lung Res* 21:791-808.

Hayashi, H., Abdollah, S., Qiu, Y., Cai, J., Xu, Y.Y., Grinnell, B.W., Richardson, M.A., Topper, J.N., Gimbrone, M.A., Jr., Wrana, J.L., Falb, D. 1997. The MAD-related protein Smad7 associates with the TGFbeta receptor and functions as an antagonist of TGFbeta signaling. *Cell*  89:1165-1173.

Hieshima, K., Imai, T., Baba, M., Shoudai, K., Ishizuka, K., Nakagawa, T., Tsuruta, J., Takeya, M., Sakaki, Y., Takatsuki, K., Miura, R., Opdenakker, G., Van Damme, J., Yoshie, O., Nomiyama, H. 1997. A novel human CC chemokine PARC that is most homologous to macrophage-inflammatory protein-1 alpha/LD78 alpha and chemotactic for T lymphocytes, but not for monocytes. *J Immunol* 159:1140-1149.

Hirsch, C.S., Toossi, Z., Johnson, J.L., Luzze, H., Ntambi, L., Peters, P., McHugh, M., Okwera, A., Joloba, M., Mugyenyi, P., Mugerwa, R.D., Terebuh, P., Ellner, J.J. 2001. Augmentation of apoptosis and interferon-γ production at sites of active *Mycobacterium tuberculosis* infection in human tuberculosis. *J Infect Dis* 183:779-788.

Hirsch, C.S., Toossi, Z., Othieno, C., Johnson, J.L., Schwander, S.K., Robertson, S., Wallis, R.S., Edmonds, K., Okwera, A., Mugerwa, R., Peters, P., Ellner, J.J. 1999. Depressed T-cell interferon-gamma responses in pulmonary tuberculosis: analysis of underlying mechanisms and modulation with therapy. *J Infect Dis*  180:2069-2073.

Holland SM, Eisenstein EM, Kuhns DB, Turner ML, Fleisher TA, Strober W, and Gallin

JI. 1994;Treatment of refractory disseminated nontuberculous mycobacterial infection with interferon gamma. *N Engl J Med.* 330: 1348-1355.

Holmes, A., Abraham, D.J., Sa, S., Shiwen, X., Black, C.M., Leask, A. 2001. CTGF and SMADs, maintenance of scleroderma phenotype is independent of SMAD signaling. *J Biol Chem*  276:10594-10601.

Honda, Y., Rogers, L., Nakata, K., Zhao, B-Y., Pine, R., Nakai, Y., Kurosu, K., Rom, W.N., Weiden, M. 1998. Type I interferon induces inhibitory 16-kD CCAAT/Enhancer Binding Protein (C/EBP)β, repressing the HIV-1 long terminal repeat in macrophages: Pulmonary tuberculosis alters C/EBP expression, enhancing HIV-1 replication. 1998. *J Exp Med* 188:1255-1265.

Hong Kong Chest Service / Tuberculosis Research Centre Madras / British Medical Research Council. 1991. A controlled clinical comparison of 6 and 8 months of antituberculosis chemotherapy in the treatment of patients with silicotuberculosis in Hong Kong. *Am Rev Respir Dis* 143:262-267,

Igarashi, A., Okochi, H., Bradham, D.M., Grotendorst, G.R. 1993. Regulation of connective tissue growth factor gene expression in human skin fibroblasts and during wound repair. *Mol Biol Cell*  4:637-45.

Ignotz, R.A., Endo, T., Massague, J. 1987. Regulation of fibronectin and type I collagen mRNA levels by transforming growth factor-beta. *J Biol Chem*  262:6443-6446.

Ihle, J.N. 1996. STATs: signal transducer and activators of transcription. *Cell* 84:331-334.

Improta, T., Pine, R., Pfeffer, L. 1992. Interferon-gamma potentiates the antiviral response and transcriptional activation induced by interferon-alpha in U937 cells. *J Interferon Cytokine Res* 12:87-94.

Itoh, S., Itoh, F., Goumans, M.J., Ten Dijke, P. 2000. Signaling of transforming growth factor-beta family members through Smad proteins. *Eur J Biochem*  267:6954-6967.

Jaffe, H.A., Buhl, R., Mastrangeli, A., Holroyd, K.J., Saltini, C., Czerski, D., Jaffe, H.S., Kramer, S., Sherwin, S., Crystal, R.G. 1991. Local activation of mononuclear phagocytes by delivery of an aerosol of recombinant interferon-γ to the human lung. *J Clin Invest* 88:297-302.

Jagirdar, J., Begin, R., Dufresne, A., Goswami, S., Lee, T.C., Rom, W.N. 1996. Transforming growth factor-β (TGF-β) in silicosis. *Am J Respir Crit Care Med* 154:1076-1081.

Jouanguy, E., Altare, F., Lamhamedi, S., Revy, P., Emile, J-F, Newport, M., Levin, M., Blanche, S., Seboun, E., Fischer, A. et al. 1996. Interferon-γ receptor deficiency in an infant with fatal Bacille Calmette-Guérin infection. *N Engl J Med* 335:1956-1961.

Jouanguy, E., Dupuis, S., Pallier, A., Doffinger, R., Fondaneche, M-C., Fieschi, C., Lamhamedi-Cherradi, S., Altare, F., Emile, J-F, Lutz, P., et al. 2000. In a novel form of IFN-γ receptor 1 deficiency, cell surface receptors fail to bind IFN-γ. *J Clin Invest* 105:1429-1436.

Kamijo, R., Shapiro, D., Le, J., Huang, S., Aguet, M., Vilcek, J. 1993. Generation of nitric oxide and induction of major histocompatibility complex class II antigen in macrophages from mice lacking the interferon γ receptor*. Proc Natl Acad Sci USA*. 90:6626-6630.

Kamijo, R., Harada, H., Matsuyama, T., Bosland, M., Gerecitano, J., Shapiro, D., Le, J., Koh, S.I., Kimura, T., Green, S.J. et al. 1994. Requirement for transcription factor IRF-1 in NO synthase induction in macrophages. *Science* 263:1612-1615.

Kamijo, R., Le, J., Shapiro, D., Havell, E.A., Huang, S., Aguet, M., Bosland, M., Vilcek, J. 1993. Mice that lack the interferon-γ receptor have profoundly altered responses to infection with Bacillus Calmette-Guérin and subsequent challenge with lipopolysaccharide. *J Exp Med* 178:1435-1440.

Kaminski, N., Allard, J.D., Pittet, J.F., Zuo, F., Griffiths, M.J.D., Morris, D., Huang, X, Sheppard, D., Heller, R.A. 2000. Global analysis of gene expression in pulmonary fibrosis reveals distinct programs regulating lung inflammation and fibrosis. *Proc Natl Acad Sci USA* 97:1778-1783.

Kavsak, P., Rasmussen, R.K., Causing, C.G., Bonni, S., Zhu, H., Thomsen, G.H.,Wrana, J.L. 2000. Smad7 binds to Smurf2 to form an E3 ubiquitin ligase that targets the TGF beta receptor for degradation. *Mol Cell*  6:1365-1375.

Kimura, T., Nakayama, K., Penninger, J., Kitagawa, M., Harada, H., Matsuyama, T., Tanaka, N., Kamijo, R., Vilcek, J., Mak, T.W., et al. 1994. Involvement of the IRF-1 transcription factor in antiviral responses to interferons. *Science* 264:1921-1924.

Kitamura, M. 1997. Identification of an inhibitor targeting macrophage production of monocyte chemoattractant protein-1 as TGF-beta 1. *J Immunol*  159:1404-11.

Klingler, K., Tchou-Wong, K.M., Brandli, O., Aston, C., Kim, R., Chi, C., Rom, W.N. 1997. Effects of mycobacteria on regulation of apoptosis in mononuclear phagocytes. *Infect Immun* 65:5272-5278.

Kostrikis, L.G., Tyagi, S., Mhlanga, M.M., Ho, D.D., Kramer, F.R. 1998. Spectral genotyping of human alleles. *Science* 279:1228-1229.

Krupsky, M., Fine, A., Kuang, P.P., Berk, J.L.,Goldstein, R.H. 1996. Regulation of type I collagen production by insulin and transforming growth factor-beta in human lung fibroblasts. *Connect Tissue Res*  34:53-62.

Lasky, J.A., Ortiz, L.A., Tonthat, B., Hoyle, G.W., Corti, M., Athas, G., Lungarella, G., Brody, A., Friedman, M. 1998. Connective tissue growth factor mRNA expression is upregulated in bleomycin-induced lung fibrosis. *Am J Physiol*  275:L365-371.

Law, K.F., Jagirdar, J., Weiden, M., Bodkin, M., Rom, W.N. 1996. Tuberculosis in HIV-positive patients: cellular response and immune activation in the lung. *Am J Respir* *Crit Care Med* 153:1377-1384.

Lee, Y.J., Han, Y., Lu, H.T., Nguyen, V., Qin, H., Howe, P.H., Hocevar, B.A., Boss, J.M., Ransohoff, R.M., Benveniste, E.N. 1997. TGF-beta suppresses IFN-gamma induction of class II MHC gene expression by inhibiting class II transactivator messenger RNA expression. *J Immunol*  158:2065-2075.

Levy, D.E., Kessler, D.S., Pine, R., Darnell, J.E., Jr. 1989. Cytoplasmic activation of ISGF3, the positive regulator of interferon-γ-stimulated transcription, reconstituted *in vitro. Genes Dev* 3:1362-1371.

Levy, D.E., Kessler, D.S., Pine, R., Reich, N., Darnell, J.E., Jr. 1988. Interferon-induced nuclear factors that bind a shared promoter element correlate with positive and negative transcriptional control*. Genes Dev* 2:383-393.

Lohoff, M., Ferrick, D., Mittrucker, H.-W., Duncan, G.S., Bischof, S., Rollinghoff, M., Mak, T.W. 1997. Interferon regulatory factor-1 is required for a T helper 1 immune response *in vivo*. *Immunity* 6:681-689.

Ludviksson, B.R., Seegers, D., Resnick, A.S., Strober, W. 2000. The effect of TGF-beta1 on immune responses of naive versus memory CD4+ Th1/Th2 T cells. *Eur J Immunol*  30:2101-2111.

Lukey, P.T., Latouf, S.E., Ress, S.R. 1996. Memory lymphocytes from tuberculous effusions: purified protein derivative (PPD) stimulates accelerated activation marker expression and cell cycle progression. *Clin Exp Immunol* 104:412-418.

MacMicking, J.D., North, R.J., LaCourse, R., Mudgett, J.S., Shah, S.K., Nathan, C.F. 1997. Identification of nitric oxide synthase as a protective locus against tuberculosis. *Proc Natl Acad Sci USA*. 94:5243-5248.

Majumdar, S., Li, D., Ansari, T., Pantelidis, P., Black, C.M., Gizycki, M., du Bois, R.M., Jeffery, P.K. 1999. Different cytokine profiles in cryptogenic fibrosing alveolitis and fibrosing alveolitis associated with systemic sclerosis: a quantitative study of open lung biopsies. *Eur Respir J* 14:251-257.

Margolin, J. 2001. From comparative and functional genomics to practical decisions in the clinic: a view from the trenches. *Genome Res*  11:923-5.

Martin RJ, Boguniewicz M, Henson JE, Celniker AC, Williams M, Giorno RC, and

Leung DYM.1993. The effects of inhaled interferon gamma in normal human airways. *Am Rev Respir Dis*. 148: 1677-1682.

Massague, J. and Chen, Y.G. 2000. Controlling TGF-beta signaling. *Genes Dev*  14:627-644.

Massague, J. and Wotton, D. 2000. Transcriptional control by the TGF-beta/Smad signaling system. *Embo J*  19:1745-1754.

Matsuyama, T., Kimura, T., Kitagawa, M., Pfeffer, K., Kawakami, T., Watanabe, N., Kündig, T.M., Amakawa, R., Kishihara, K., Wakeham, A., et al. Targeted disruption of IRF-1 or IRF-2 results in abnormal type I IFN gene induction and aberrant lymphocyte development. Cell 1993;75:83-97.

Meraz, M.A., White, J.M., Sheehan, K.C.F., Bach, E.A., Rodig, S.J., Dighe, A.S., Kaplan, D.H., Riley, J.K., Greenlund, A.C., Campbell, D., et al. 1996. Targeted disruption of the Stat 1 gene in mice reveals unexpected physiologic specificity in the JAK-STAT signaling pathway. *Cell* 84:431-442.

Mosiman, V.L., Patterson, B.K., Canterero, L., Goolsby, C.L. 1997. Reducing cellular autofluorescence in flow cytometry: an in situ method. *Cytometry* 30:151-156.

Munger, J.S., Huang, X., Kawakatsu, H., Griffiths, M.J., Dalton, S.L., Wu, J., Pittet, J.F., Kaminski, N., Garat, C., Matthay, M.A., Rifkin, D.B., Sheppard, D. 1999. The integrin alpha v beta 6 binds and activates latent TGF beta 1: a mechanism for regulating pulmonary inflammation and fibrosis. *Cell*  96:319-328.

Munger, J.S., Huang, X., Kawakatsu, H., Griffiths, M.J.D., Dalton, S.L., Wu, J., Pittet, J.-F., Kaminski, N., Garat, C., Matthay, M.A., Rifkin, D.B., Sheppard, D. 1999. The integrin αvβ6 binds and activates latent TGFβ1: a mechanism for regulating pulmonary inflammation and fibrosis. *Cell* 96:319-328.

Murray, C.J.L. and Salomon, J.A. 1998. Modeling the impact of global tuberculosis control strategies. *Proc Natl Acad Sci USA*. 95:13881-13886.

Nakao, A., Afrakhte, M., Moren, A., Nakayama, T., Christian, J.L., Heuchel, R., Itoh, S., Kawabata, M., Heldin, N.E., Heldin, C.H., Ten Dijke, P. 1997. Identification of Smad7, a TGFbeta-inducible antagonist of TGF-beta signalling. *Nature*  389:631-635.

Nakata, K., Rom, W.N., Honda, Y., Condos, R., Kanegasaki, S., Cao, Y., Weiden, M. 1997. *M. tuberculosis* enhances human immunodeficiency virus-1 replication in the lung. *Am J Resp Crit Care Med* 155:996-1003.

Nandan, D. and Reiner, N.E. 1997. TGF-beta attenuates the class II transactivator and reveals an accessory pathway of IFN-gamma action. *J Immunol*  158:1095-1101.

Newport, M.J., Huxley, C.M., Huston, S., Hawrylowicz, C.M., Oostra, B.A., Williamson, R., Levin, M. 1996. A mutation in the interferon-γ receptor gene and susceptibility to mycobacterial infection. *N Engl J Med* 335:1941-1949.

Nicholson, S., Bonecini-Almeida, M.G., Silva, J.R.L., et al. 1996. Inducible nitric oxide synthase in pulmonary alveolar macrophages from patients with tuberculosis. *J Exp Med* 183:2293-2302.

Nussler, A. K. and Billiar, T. R.. 1993. Inflammation, immunoregulation, and inducible nitric oxide synthase. J Leukoc Biol 54:171-178.

Ogasawara, K., Hida, S., Azimi, N., Tagaya, Y., Sato, T., Yokochi-Fukuda, T., Waldmann, T.A., Taniguchi, T., Taki, S. 1998. Requirement for IRF-1 in the microenvironment supporting development of natural killer cells. *Nature* 391:700-703.

Ohteki, T., Yoshida, H., Matsuyama, T., Duncan, G.S., Mak, T.W., Ohashi, P.S. 1998. The Transcription Factor Interferon Regulatory Factor 1†(IRF-1) Is Important during the Maturation of Natural Killer 1.1+ T Cell Receptor-alpha /beta + (NK1+ T) Cells, Natural Killer Cells, and Intestinal Intraepithelial T Cells. *J Exp Med* 187:967-972.

O'reilly, M.S., Boehm, T., Shing, Y., Fukai, N., Vasios, G., Lane, W.S., Flynn, E., Birkhead, J.R., Olsen, B.R., Folkman, J. 1997. Endostatin: an endogenous inhibitor of angiogenesis and tumor growth. *Cell*  88:277-285.

Othieno, C., Hirsch, C.S., Hamilton, B.D., Wilkinson, K., Ellner, J.J., Toossi, Z. 1999. Interaction of Mycobacterium tuberculosis-induced transforming growth factor beta1 and interleukin-10. *Infect Immun*  67:5730-5735.

Passmore, J.S., Glashoff, R.H., Lukey, P.T., Ress, S.R. 2001. Granule-dependent cytolysis of *Mycobacterium tuberculosis*-infected macrophages by human gd^+^ T cells has no effect on intracellular mycobacterial viability. *Clin Exp Immunol* 125:1-9.

Piek, E., Heldin, C.H., Ten Dijke, P. 1999. Specificity, diversity, and regulation in TGF-beta superfamily signaling. *Faseb J*  13:2105-2124.

Piek, E., Ju, W.J., Heyer, J., Escalante-Alcalde, D., Stewart, C.L., Weinstein, M., Deng, C., Kucherlapati, R., Bottinger, E.P., Roberts, A.B. 2001. Functional characterization of transforming growth factor beta signaling in Smad2- and Smad3-deficient fibroblasts. *J Biol Chem*  276:19945-19953.

Pine, R. Constitutive expression of an ISGF2/IRF1 transgene leads to interferon-independent activation of interferon-inducible genes and resistance to virus infection. 1992. *J Virol* 66:4470-4478.

Pine, R., Canova, A., Schindler, C. 1994. Tyrosine phosphorylated p91 binds to a single element in the ISGF2/IRF-1 promoter to mediate induction by IFN α and IFN γ, and is likely to autoregulate the p91 gene. *Embo J*  13:158-167.

Pine, R., Decker, T., Kessler, D.S., Levy, D.E., Darnell, J.E., Jr. 1990. Purification and cloning of interferon-stimulated gene factor 2 (ISGF2): ISGF2 (IRF-1) can bind to the promoters of both beta interferon- and interferon-stimulated genes but is not a primary transcriptional activator of either. *Mol Cell Biol*  10:2448-2457.

Piskurich, J.F., Wang, Y., Linhoff, M.W., White, L.C., Ting, J.P. 1998. Identification of distinct regions of 5' flanking DNA that mediate constitutive, IFN-gamma, STAT1, and TGF-beta-regulated expression of the class II transactivator gene. *J Immunol*  160:233-240.

Raghu G, Brown KK, Bradford WZ, Starko K, Noble PW, Schwartz DA, King TE Jr;

Idiopathic Pulmonary Fibrosis Study Group A placebo-controlled trial of interferon gamma-1b in patients with idiopathic pulmonary fibrosis. N Engl J Med. 2004;350(2):125-33.

Raju, B., Tung, C.F., Cheng, D., Yousefzadeh, N., Condos, R., Rom, W.N., Tse, D.B. 2001. *In situ* activation of helper T cells in the lung. *Infect immune* 69:4790-4798.

Ramos, C., Montano, M., Garcia-Alvarez, J., Ruiz, V., Uhal, B.D., Selman, M., Pardo, A. 2001. Fibroblasts from idiopathic pulmonary fibrosis and normal lungs differ in growth rate, apoptosis, and tissue inhibitor of metalloproteinases expression. *Am J Respir Cell Mol Biol*  24:591-598.

Reich, N., Evans, B., Levy, D., Fahey, D., Knight, E., Jr, Darnell, J.E., Jr. 1987. Interferon-induced transcription of a gene encoding a 15-kDa protein depends on an upstream enhancer element. *Proc Natl Acad Sci USA*. 84:6394-6398.

Roberts, A.B. 1999. TGF-beta signaling from receptors to the nucleus. *Microbes Infect*  1:1265-1273.

Rojas, R.E., Balaji, K.N., Subramanian, A., Boom, W.H. 1999. Regulation of human CD4(+) alphabeta T-cell-receptor-positive (TCR(+)) and gammadelta TCR(+) T-cell responses to Mycobacterium tuberculosis by interleukin-10 and transforming growth factor beta. *Infect Immun*  67:6461-6472.

Rom, W.N., Schluger, N., Law, K., Condos, R., Zhang, Y., Weiden, M., Harkin, T., Tchou-Wong, K.-M. 1995. Human host response to *Mycobacterium tuberculosis*. *Schweiz Med Wochenschr* 125:2178-2185.

Rosenberger, C.M., Scott, M.G., Gold, M.R., Hancock, R.E., Finlay, B.B. 2000. Salmonella typhimurium infection and lipopolysaccharide stimulation induce similar changes in macrophage gene expression. *J Immunol*  164:5894-904.

Rosendahl, A., Checchin, D., Fehniger, T.E., ten Dijke, P., Heldin, C.-H., Sideras, P. 2001. Activation of the TGF-β/activin-Smad2 pathway during allergic airway inflammation. *Am J Respir Cell Mol Biol* 25:60-68.

Salkowski, C.A., Barber, S.A., Detore, G.R., Vogel, S.N. 1996. Differential dysregulation of nitric oxide production in macrophages with targeted disruptions in IFN regulatory factor-1 and –2 genes. *J Immunol* 156:3107-3110.

Salkowski, C.A., Kopydlowski, K., Blanco, J., Cody, M.J., McNally, R., Vogel, S.N. 1999. IL-12 is dysregulated in macrophages from IRF-1 and IRF-2 knockout mice. *J* *Immunol* 163:1529-1536.

Sallusto, F., Lenig, D., Mackay, C.R., Lanzavecchia, A. 1998. Flexible programs of chemokine receptor expression on human polarized T helper 1 and 2 lymphocytes. *J Exp Med* 187:875-883.

Scanga, C.A., Mohan, V.P., Yu, K., Joseph, H., Tanaka, K., Chan, J., Flynn, J.L. 2000. Depletion of CD4(+) T cells causes reactivation of murine persistent tuberculosis despite continued expression of interferon gamma and nitric oxide synthase 2. *J Exp Med* 192:347-358.

Schluger, N.W., Huberman, R., Holzman, R., Rom, W.N., Cohen, D.I. 1999. Screening for infection and disease as a tuberculosis control measure among indigents in New York City, 1994-1997. *Int J Tuberculosis Lung Dis* 3:281-286.

Seder, R.A., Marth, T., Sieve, M.C., Strober, W., Letterio, J.J., Roberts, A.B.Kelsall, B. 1998. Factors involved in the differentiation of TGF-beta-producing cells from naive CD4+ T cells: IL-4 and IFN-gamma have opposing effects, while TGF-beta positively regulates its own production. *J Immunol*  160:5719-5728.

Selwyn, P.A., Hartel ,D., Lewis, V.A., Schoenbaum, E.E., Vermund, S.H., Klein, R.S., Walker, A.T., Freidland, G.H. 1989. A prospective study of the risk of tuberculosis among intravenous drug users with human immunodeficiency virus infection. *N Engl J Med* 320:545-550.

Sime, P.J., Xing, Z., Graham, G.L., Csaky, K.G., Gauldie, J. 1997. Adenovector-mediated gene transfer of active transforming growth factor β1 induces prolonged severe fibrosis in rat lung. *J Clin Invest* 100:768-776.

Strieter RM, Starko KM, Enelow RI, Noth I, Valentine VG. Effects of interferon

gamma1b on biomarker expression in idiopathic pulmonary fibrosis patients. *Am J Respir Crit Care Med.* 2004; 170:133-140

Tajirian, T., Dennis, J.W., Swallow, C.J. 2000. Regulation of human monocyte proMMP-9 production by fetuin, an endogenous TGF-beta antagonist. *J Cell Physiol*  185:174-83.

Thiel, A., Schmitz, J., Miltenyi, S., Radbruch, A. 1997. CD45RA-expressing memory/efector Th cells committed to production of interferon-gamma lack expression of CD31. *Immunol Lett* 57:189-192.

Tomioka, H., Shimizu, T., Maw, W.W., Ogasawara, K. 2000. Roles of tumour necrosis factor-alpha (TNF-alpha), transforming growth factor-beta (TGF-beta), and IL-10 in the modulation of intercellular adhesion molecule-1 (ICAM-1) expression by macrophages during mycobacterial infection. *Clin Exp Immunol*  122:335-342.

Toosi, Z., Nicolacakis, K., Xia, L., Ferrari, N.A., Rich, E.A. 1997. Activation of latent HIV-1 by Mycobacterium tuberculosis and its protein purified derivative in alveolar macropjhages from HIV-infected individuals in vitro. *J Acquir Immune Defic Syndr Hum Retrovirol* 15:325-331.

Toossi, Z., Hirsch, C.S., Hamilton, B.D., Knuth, C.K., Friedlander, M.A.Rich, E.A. 1996. Decreased production of TGF-beta 1 by human alveolar macrophages compared with blood monocytes. *J Immunol*  156:3461-3468.

Toossi, Z. and Ellner, J.J. 1998. The role of TGF beta in the pathogenesis of human tuberculosis. *Clin Immunol Immunopathol*  87:107-114.

Tredget, E.E., Wang, R., Shen, Q., Scott, P.G., Ghahary, A. 2000. Transforming growth factor-beta mRNA and protein in hypertrophic scar tissues and fibroblasts: antagonism by IFN-alpha and IFN-gamma in vitro and in vivo. *J Interferon* (TGF beta) causes a persistent increase in steady-state amounts of type I and type III collagen and fibronectin mRNAs in normal human dermal fibroblasts. *Bioche* 597-604.

Tyagi, S. and Kramer, F.R. 1996. Molecular beacons: probes that fluoresce upon hybridization. *Nat Biotechnol* 14:303-308.

Ulloa, L., Doody, J., Massague, J. 1999. Inhibition of transforming growth factor-beta/SMAD signalling by the interferon-gamma/STAT pathway. *Nature*  397:710-713.

Varga, J., Rosenbloom, J., Jimenez, S.A. 1987. Transforming growth factor beta (TGF beta) causes a persistent increase in steady-state amounts of type I and type III collagen and fibronectin mRNAs in normal human dermal fibroblasts. *Biochem J*  247:597-604.

Vet, J.A., Majithia, A.R., Marras, S.A., Tyagi, S., Dube, S., Poiesz, B.J., Kramer, F.R. 1999. Multiplex detection of four pathogenic retroviruses using molecular beacons. *Proc Natl Acad Sci USA*  96:6394-6399.

Von Gersdorff, G., Susztak, K., Rezvani, F., Bitzer, M., Liang, D., Bottinger, E.P. 2000. Smad3 and Smad4 mediate transcriptional activation of the human Smad7 promoter by transforming growth factor beta. *J Biol Chem*  275:11320-11326.

Wahl, S.M., Allen, J.B., Weeks, B.S., Wong, H.L., Klotman, P.E. 1993. Transforming growth factor beta enhances integrin expression and type IV collagenase secretion in human monocytes. *Proc Natl Acad Sci USA*  90:4577-4581.

Wahl, S.M., Mccartney-Francis, N., Allen, J.B., Dougherty, E.B., Dougherty, S.F. 1990. Macrophage production of TGF-beta and regulation by TGF-beta. *Ann N Y Acad Sci*  593:188-196.

Weiden, M., Tanaka, N., Qiao, Y., Zhao, B.Y., Honda, Y., Nakata, K., Canova, A., Levy, D.E., Rom, W.N., Pine, R. 2000. Differentiation of Monocytes to Macrophages Switches the *Mycobacterium tuberculosis* Effect on HIV-1 Replication from Stimulation to Inhibition: Modulation of Interferon Response and CCAAT/Enhancer Binding Proteinβ Expression. *J Immunol* 165:2028-2039.

Whalen, C., Horsburgh, C.R., Hom, D., Lahart, C., Simberkoff, M., Ellner, J. 1995 Accelerated course of human immunodeficiency virus infection after tuberculosis. *Am J Respir Crit Care Med* 151:129-135.

White, LC., Wright, K.L., Felix, N.J., Ruffner, H., Reis, L.F.L., Pine, R., Ting, J.P. 1996. Regulation of LMP2 and TAP1 genes by IRF-1 explains the paucity of CD8+ T cells in IRF-1-/- mice. *Immunity* 5:365-376.

Worgall, S., Connor, R., Kaner, R.J., Fenamore, E., Sheridan, K., Singh, R., Crystal, R.G. 1999. Expression and use of human immunodeficiency virus type 1 coreceptors by human alveolar macrophages. *J Virol* 73:5865-5874.

World Health Organization. Groups at Risk. WHO Report on the Tuberculosis Epidemic. Geneva, 1996.

Wotton, D. and Massague, J. 2001. Smad transcriptional corepressors in TGF beta family signaling. *Curr Top Microbiol Immunol*  254:145-164.

Xiao, B.G., Zhang, G.X., Ma, C.G., Link, H. 1996. Transforming growth factor-beta 1 (TGF-beta1)-mediated inhibition of glial cell proliferation and down-regulation of intercellular adhesion molecule-1 (ICAM-1) are interrupted by interferon-gamma (IFN-gamma). *Clin Exp Immunol*  103:475-481.

Yuan, W., Yufit, T., Li, L., Mori, Y., Chen, S.J., Varga, J. 1999. Negative modulation of alpha1(I) procollagen gene expression in human skin fibroblasts: transcriptional inhibition by interferon-gamma. *J Cell Physiol*  179:97-108.

Zawel, L., Dai, J.L., Buckhaults, P., Zhou, S., Kinzler, K.W., Vogelstein, B., Kern, S.E. 1998. Human Smad3 and Smad4 are sequence-specific transcription activators. *Mol Cell*  1:611-617.

Zhang, M., Gong, J., Presky, D.H., Xue, W.Barnes, P.F. 1999. Expression of the IL-12 receptor beta 1 and beta 2 subunits in human tuberculosis. *J Immunol*  162:2441-2447.

Zhang, W., Ou, J., Inagaki, Y., Greenwel, P., Ramirez, F. 2000. Synergistic cooperation between Sp1 and Smad3/Smad4 mediates transforming growth factor beta1 stimulation of alpha 2(I)-collagen (COL1A2) transcription. *J Biol Chem*  275:39237-39245.

Zhang, Y., Nakata, K., Weiden, M., Rom, W. 1995. *Mycobacterium tuberculosis* enhances HIV-1 replication by transcrptional activation at the long terminal repeat. *J Clin Invest* 95:2324.

Zierski, M., Bek, E., Long, M.W., Snider, D.E., Jr. 1980. Short-course (6 month) cooperative tuberculosis study in Poland: results 18 months after completion of treatment. *Am Rev Respir Dis* 122:879-889.

Ziesche, R., Hofbauer, E., Wittmann, K., Petkov, V., Block, L.-H. 1999. A Preliminary study of long-term treatment with interferon gamma-1b and low-dose prednisolone in patients with idiopathic pulmonary fibrosis. *N Engl J Med* 341:1264-1269.

Zimmerman, C.M. and Padgett, R.W. 2000. Transforming growth factor beta signaling mediators and modulators. *Gene*  249:17-30.

Zohlnhofer, D., Richter, T., Neumann, F., Nuhrenberg, T., Wessely, R., Brandl, R., Murr, A., Klein, C.A.,Baeuerle, P.A. 2001. Transcriptome analysis reveals a role of interferon-gamma in human neointima formation. *Mol Cell*  7:1059-1069.

**H. CONSORTIUM/CONTRACTUAL ARRANGEMENTS.**

The Division of Pulmonary and Critical Care Medicine at NYU School of Medicine is the parent applicant organization, with TB patients enrolled from the Bellevue Chest Service and studied on the General Clinical Research Center (GCRC). The subcontract to the University of Cape Town enables us to reach the sample size of 64 study subjects and provides a clinical research collaboration. The cellular immunology studies using flow cytometry will be performed in the setting of an ongoing collaboration between the GCRC and the Center for AIDS Research (CFAR). The subcontract with the Public Health Research Institute (PHRI) on TGF-β is a collaboration that was in the original RO1 HL59832, in which we studied the IFN-γ signaling system.

Letters confirming collaboration from Dr. Ress (Consortium I) and Dr. Pine (Consortium II) are included below.

**I. CONSULTANTS.**

Dr. Munger’s role will be to consult with us on TGF-β biology. He has developed the assay for active TGF-β. He will advise us on our assays and scientific approaches. He has a NIH RO1 grant on TGF-β activation. A Letter from Dr. John Munger confirming his role on the project is included below.
